# Psycho-Emotional Profile and Disease Burden in Wild-Type Transthyretin Amyloidosis

**DOI:** 10.3390/jcm15166348

**Published:** 2026-08-17

**Authors:** Sandra Michaela Ihne-Schubert, Marcel Weidgans, Annelie Neubauer, Maria Leberzammer, Moritz Schütt, Aikaterini Papagianni, Caroline Morbach, Marc Becker, Thomas Wehler, Hermann Einsele, Claudia Sommer, Andreas Geier, Stefan Störk, Silke Neuderth

**Affiliations:** 1 Department of Internal Medicine IV, University Hospital of Gießen and Marburg, Justus-Liebig-University Gießen, 35392 Gießen, Germany; 2 Interdisciplinary Amyloidosis Center of Northern Bavaria, University Hospital of Würzburg, 97080 Würzburg, Germanystoerk_s@ukw.de (S.S.); 3 Department of Internal Medicine II, University Hospital of Würzburg, 97080 Würzburg, Germany; 4 CIRCLE—Centre for Innovation Research, Lund University, Box 118, 221 00 Lund, Sweden; 5 Department of Anaesthesia, Intensive Care Medicine and Pain Medicine, Clinical Division of General Anaesthesia and Intensive Care Medicine, Medical University of Vienna, 1090 Vienna, Austria; 6 Department of Anesthesiology, Regionale Kliniken Holding (RKH) Klinikum Ludwigsburg, 71640 Ludwigsburg, Germany; 7 Department of Neurology, University Hospital Ulm, 89081 Ulm, Germany; 8 Department of Neurology, University Hospital Würzburg, 97080 Würzburg, Germany; 9 Department of Clinical Research and Epidemiology, Comprehensive Heart Failure Center (CHFC), University Hospital Würzburg, 97080 Würzburg, Germany; 10 Department of Internal Medicine I, University Hospital of Würzburg, 97080 Würzburg, Germany; 11 Institute for Applied Social Sciences (IFAS), Technical University of Applied Sciences Würzburg-Schweinfurt (THWS), 97070 Würzburg, Germany; silke.neuderth@thws.de

**Keywords:** ATTRwt amyloidosis, psychological burden, distress, fear of progression, fatigue, patient-reported outcomes

## Abstract

**Background/Objectives:** Patients with transthyretin amyloidosis frequently experience reduced quality of life, distress, anxiety and depression. But the evidence is inconsistent and the constructs are often not clearly separated. This study assessed quality of life and mental health symptoms in wild-type transthyretin amyloidosis (ATTRwt). **Methods:** Within the amyloidosis cohort study AmyKoS, 113 patients with confirmed ATTRwt amyloidosis completed a structured survey between July 2020 and February 2024, assessing quality of life (Short Form (36) Health Survey, SF-36), distress (Distress Thermometer), anxiety (Generalized Anxiety Disorder-7, GAD-7), depression (Patient Health Questionnaire-9, PHQ-9), fear of progression (Fear of Progression Questionnaire, short form; PA-F-KF), fatigue and dyspnea. **Results:** Of 113 patients (age 78.95 ± 5.90 years, 14.2% female), 88.4% reported restricted quality of life and 62.7% screened positive for distress. Overall, 26.4% screened positive for anxiety and/or depression and 9.2% for fear of progression. Both SF-36 summary scales were reduced to a comparable degree relative to the age-matched general population: physical component summary 36.9 ± 9.8 versus 43.5 ± 11.8 and mental component summary 46.8 ± 9.1 versus 52.2 ± 10.3 (both *p* < 0.001, Cohen’s d = −0.59 and −0.54). Notably, 59.4% of distress-positive patients screened positive for neither anxiety nor depression, and fatigue was the key predictor. **Conclusions:** In ATTRwt amyloidosis, both dimensions of health-related quality of life are substantially impaired. Stepwise screening should be routine, and many burdened patients are likely to benefit primarily from symptom control and rehabilitation rather than psychiatric treatment.

## 1. Introduction

Transthyretin (ATTR) amyloidosis is caused by the dissociation of the transthyretin tetramer into monomers and dimers that misfold and aggregate as amyloid fibrils and deposit as insoluble amyloid in the extracellular space of the heart, the peripheral nerves and connective tissue [1,2]. Two forms are distinguished: The wild-type form (ATTRwt) arises without a genetic variant, typically affects the elderly and predominantly men, and results in a cardiac phenotype with secondary neurological involvement [1,2]. In contrast, the variant form (ATTRv) results from pathogenic mutations in the TTR gene, occurs with two peaks in early (25–40 years) and in later adulthood (55–75 years) and shows a broader phenotypic spectrum, including isolated cardiac or neurological manifestations or mixed phenotypes depending on the underlying variant [1,2].

ATTR amyloidosis has long been considered a rare disease, but in particular, the recognized frequency of the wild-type form has increased substantially since the introduction of non-invasive diagnosis by bone scintigraphy, approval of disease-modifying drugs and improved awareness among health care providers [3,4,5]. According to Lauppe et al. [6], the prevalence of cardiac ATTR amyloidosis ranges between 1.4 and 5 per 100,000 according to national health registries in Northern Europe, so the disease is now considered an underdiagnosed rather than a genuinely rare cause of heart failure.

Clinically, cardiac involvement in ATTRwt amyloidosis (ATTRwt-CA) presents as restrictive cardiomyopathy, whereas neurological presentation includes progressive sensorimotor and autonomic polyneuropathy [1,2]. Red flags such as carpal tunnel syndrome, lumbar spinal stenosis and spontaneous tendon rupture frequently precede the cardiac diagnosis by several years [1,2]. Untreated, the disease is progressive and fatal [1,2]. Since the approval of TTR stabilizers such as tafamidis and, subsequently, of gene-silencing agents, the prognosis has improved considerably, which has shifted attention towards outcomes that matter to patients over longer periods of survival [5].

Quality of life is substantially impaired in ATTR amyloidosis. The Italian ITALY study systematically characterized patient-reported outcome measures in cardiac ATTR amyloidosis and demonstrated relevant limitations across physical and social domains [7]. In a large cohort from a North American referral center, physical component summary scores of the SF-36 were markedly reduced across all ATTR subtypes and approached the values observed in large heart failure populations, whereas mental component summary scores remained close to population norms [8]. Focus groups have shown that patients with cardiac and with neuropathic manifestations consistently name fatigue, restricted activity and loss of independence as the most burdensome aspects of the disease [9]. These findings suggest that impairment of physical function and of daily participation is a principal mechanism through which the disease affects emotional wellbeing.

Depression is common in heart failure in general, affecting approximately one in five patients overall and rising with NYHA functional class [10], which provides the clinical context but is not specific to amyloidosis. Evidence relating specifically to ATTR amyloidosis is more limited. Aimo et al. reviewed the patient-reported outcome measures used across ATTRwt and ATTRv amyloidosis and concluded that no disease-specific instrument was available, so generic scales and instruments developed for other conditions had to be used [11]. This gap has since prompted the development and validation of the ATTR-specific quality of life questionnaire ATTR-QOL, which includes an emotional wellbeing scale [12,13], with first clinical experience reported only recently [14]. Data on anxiety and depression in ATTR amyloidosis remain heterogeneous and sometimes controversial. For example, Smorti et al. reported clinically relevant anxiety and depressive symptoms in 49% of patients [15], whereas Eldhagen et al. found no signs of depression in the large majority (89%) of their cohort [16]. Reported predictors of impaired quality of life and/or mental health conditions such as anxiety and depression include older age, female sex, more advanced disease (NYHA functional class, NAC stage), and more pronounced symptom burden [15,16,17]. Findings from the hereditary form are only partly transferable. In a Portuguese THAOS cohort with ATTRv polyneuropathy, the anxiety and depression dimension of the EQ-5D was affected in 57% of patients [18], and a caregiver survey documented higher rates of anxiety and depression among relatives of patients with ATTRv amyloidosis than among matched controls [19]. In both settings, concerns about heritability and family screening contribute to the burden, and these are absent in wild-type form. Data on fear of progression, a construct well-established in oncology and increasingly studied in chronic cardiac disease, are not available for ATTR amyloidosis at all.

Therefore, we screened patients with ATTRwt amyloidosis using questionnaires assessing quality of life, distress, anxiety, depression, fear of progression, social aspects and coping strategies and correlated the results with clinical findings. We specifically aimed to distinguish nonspecific distress, which may largely reflect physical symptom burden, from anxiety and depressive symptoms at thresholds that indicate a need for further clinical assessment.

## 2. Materials and Methods

### 2.1. Study Population and Patient Selection

Patients with proven ATTRwt amyloidosis were identified within the prospective Amyloidosis Cohort Study (AmyKoS) at the Interdisciplinary Amyloidosis Center of Northern Bavaria, Würzburg, Germany [20]. The study received approval by the Ethics Committee of the Julius-Maximilians-University Würzburg (vote #48/18) and complied with the Declaration of Helsinki. All participants provided written informed consent prior to any investigation. The clinical evaluations were performed as part of routine care and included a physical examination, detailed laboratory testing according to the local standards, standardized transthoracic echocardiography, and a 6 min walking test, as well as additional assessments according to the AmyKoS protocol (for details see Ihne-Schubert et al. 2024 [20]). During these evaluations, patients completed questionnaires to systematically assess their symptom burden regarding dyspnea (Functional Assessment of Chronic Illness Therapy-Dyspnea, FACIT-Dyspnea) and fatigue (Functional Assessment of Chronic Illness Therapy-Fatigue, FACIT-Fatigue), as well as general quality of life (SF-36), distress (Distress Thermometer), anxiety (GAD-7), depression (PHQ-9), and fear of progression (PA-F-KF), as well their social situation, needs and coping strategies (modified _A_MY-NEED_S_ questionnaire [21]).

**Setting.** Recruitment into the AmyKoS amyloidosis cohort study was started in 2018. The present analysis is a cross-sectional questionnaire survey conducted under the umbrella of AmyKoS and independent of the timing of cohort enrolment. Questionnaires were administered between July 2020 and February 2024. All questionnaires were completed during elective outpatient follow-up visits at the amyloidosis center and not during hospital admission or during episodes of cardiac decompensation.

**Inclusion and exclusion criteria.** Patients were eligible if they were AmyKoS participants with written informed consent, had confirmed systemic ATTRwt amyloidosis and were able to complete the self-administered questionnaires independently. Pre-existing psychiatric disorders and psychotropic medication were not exclusion criteria. These patients were retained in the main analysis and additionally addressed in a sensitivity analysis (Section 2.3).

**Figure 1:**
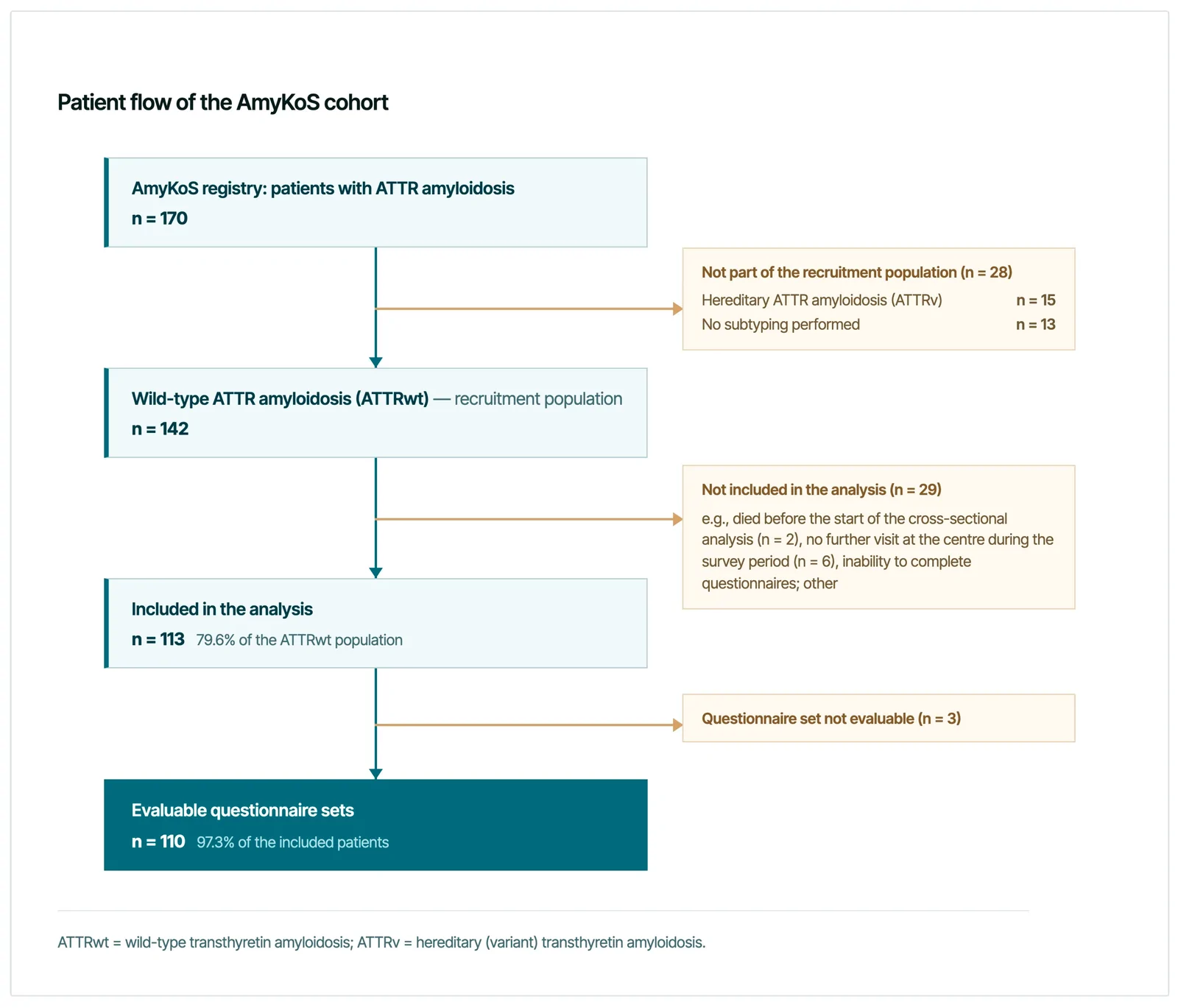
One-hundred thirteen of 142 patients with proven ATTRwt amyloidosis within the AmyKoS have been included in this analysis; 110 (97.3%) patients returned evaluable questionnaire sets.

**Cohort derivation and response.** The derivation of the analysis population is shown in Figure 1. The number of patients who were approached and those who declined was not systematically recorded. The questionnaire battery comprised the questionnaires mentioned above. Of the 113 patients included, 110 (97.3%) returned all questionnaires, whereas three patients returned only part of it. Psychometric analyses are, therefore, based on 110 patients.

**Diagnostic pathway, exclusion of a monoclonal protein disorder and genotyping.** ATTRwt amyloidosis was confirmed either by histology or bone scintigraphy in the absence of a monoclonal gammopathy, followed by genetic testing. For determination of monoclonal protein, serum electrophoresis, as well as free light chain analysis and immunofixation, were performed in serum and urine in all patients. In case of a monoclonal protein, the diagnosis was subsequently confirmed histologically, including amyloid subtyping, thereby excluding AL amyloidosis.

**Definition of organ involvement.** Cardiac involvement was defined by a positive DPD scintigraphy (Perugini grade 2 or 3) in the absence of a relevant monoclonal protein, or histological confirmation of ATTR amyloid. Neurological involvement was defined as clinically and/or electrophysiologically confirmed polyneuropathy.

### 2.2. Questionnaires

The survey included standardized assessment of quality of life by 36-Item Short Form Health Survey (SF-36), distress via Distress Thermometer, anxiety by 7-item Generalized Anxiety Disorder scale (GAD-7), depression based on 9-item Patient Health Questionnaire (PHQ-9) and fear of progression (Fear of Progression Questionnaire, short form; PA-F-KF) beyond a modified version of the _A_MY-NEED_S_ questionnaire to assess the social situation, needs and coping strategies [21].

The questionnaires were evaluated according to the respective instructions. Validated German-language versions were used for all instruments, and no disease-specific adaptation was made for ATTR amyloidosis.

Fatigue was assessed with the FACIT-Fatigue Scale version 4 in its German translation, and dyspnea with the German version of the FACIT-Dyspnea 10-item Short Form, comprising part I (severity) and part II (functional limitations) [22]. Health-related quality of life was assessed with the SF-36 version 1 in the German standard form with a four-week recall window [23,24]. Summary scales were computed using the standard norm-based algorithm. That is, z-transformation of the eight subscales against the 1998 US general population sample, weighting with the published factor coefficients, and transformation to a mean of 50 and a standard deviation of 10. This is the algorithm underlying the DEGS1 summary scales [25] and the one used by Sanchorawala et al. [8], so both comparisons are made on the same metric.

Distress was assessed with the Distress Thermometer without the accompanying problem list. A positive distress screening was defined by a Distress Thermometer value of ≥5 points. This cut-off derives from validation studies in oncological populations and has not been validated in chronic cardiovascular disease, in ATTR amyloidosis or in very elderly patients. It was retained to allow comparison with the existing literature, and its limitations in the present population are addressed in the Discussion.

For the GAD-7, PHQ-9 and PA-F-KF questionnaires, total scores were calculated. GAD-7 sum scores of up to 4 points were considered normal, with 5–9 points indicating mild anxiety, 10–14 points moderate anxiety and ≥15 points severe anxiety. PHQ-9 sum scores of up to 4 points were considered minimal depressive symptoms, while 5–9 points indicated mild depressive symptoms. Moderate depression was defined as 10–14 points, moderately severe depression as 15–19 points, and severe depression as ≥20 points. A score of ≥10 points was considered indicative of a condition requiring further clinical assessment. In addition to the sum score, the categorical diagnostic algorithm of the PHQ-9 was applied. Major depression was assumed when at least five of the nine items were endorsed as present on more than half the days, including item 1 or item 2, with item 9 counting as positive when endorsed on individual days. Other depressive syndrome was assumed when two to four items were endorsed under the same conditions. These categorical results are reported separately from the sum score categories and are not interchangeable with them.

Fear of progression was assessed with the short form of the Fear of Progression Questionnaire (PA-F-KF) [26,27,28]. The cut-off for a positive PA-F-KF result was a sum score of ≥34 points, indicating fear of progression. In addition to the sum score, the item-based classification used by Hefner et al. was applied [29]. Fear of progression was rated as moderate when at least 50% of items and as high when at least 75% of items were answered with 4 or 5, corresponding to six and nine of the twelve PA-F-KF items, respectively. The two categories are mutually exclusive. The PA-F-KF was developed and validated in oncological and other chronic disease populations and has not been validated in ATTR amyloidosis. Because the instrument may not capture concerns specific to this population, such as progressive heart failure, loss of mobility, treatment access and caregiver dependence, all findings relating to fear of progression are reported as exploratory. Because the PA-F-KF showed the highest item-level missingness of all instruments, an extrapolated sum score was calculated for questionnaires with incomplete but partially available item responses.

### 2.3. Statistical Analysis

Descriptive statistical measures include mean with standard deviation (SD), median with interquartile range (IQR), and relative frequencies. Continuous variables of the AmyKoS cohort were compared with the age-matched DEGS1 reference sample using a z-test. Reference values were derived from the DEGS1 Scientific Use File [30] for the age group 75 to 79 years and were computed as weighted estimates using the cross-sectional survey weight and accounting for clustering by sample point, following the analysis guidance of the Robert Koch Institute. Subpopulation estimation was used throughout. Subscales were compared on the 0 to 100 metric in both cohorts. For the summary scales, both cohorts were scored with the standard norm-based algorithm. That is, z-transformation of the eight subscales against the 1998 US general population sample, weighting with the published factor score coefficients and transformation to a mean of 50 and a standard deviation of 10. Since the AmyKoS cohort completed the SF-36 version 1 and DEGS1 used version 2, the version-specific US norm parameters were applied in each case, while the factor score coefficients are identical for both versions. Because the two versions differ in the response format of the role-functioning scales, small residual differences in scaling cannot be excluded. Within the cohort, groups were compared using Welch’s t-test for continuous variables and the z-test for proportions. Univariate and multivariate linear regression and probit models were used to identify predictors for quality-of-life impairment and questionnaire results. Probit models were estimated with robust standard errors. A two-sided significance level of 0.05 was applied throughout. Values with 0.05 ≤ *p* < 0.1 are reported as such and are explicitly not interpreted as evidence of an effect. Differences from the reference population were additionally expressed as Cohen’s d and interpreted against the minimal clinically important difference of the SF-36, which is generally reported as approximately 3 to 5 points for the summary scales.

**Group definition.** Patients were classified as screening positive for anxiety and/or depression if the GAD-7 sum score was ≥10 points or the PHQ-9 sum score was ≥10 points. That is, either criterion was sufficient. This composite variable is used in Tables 1 and 3 and in the probit models.

**Variables and model building.** The following variables were considered as candidate predictors, selected a priori on clinical considerations rather than on the basis of univariable significance: general condition (ECOG performance status), symptom burden (NYHA functional class, FACIT-Dyspnea Raw Dyspnea Score, FACIT-Fatigue Fatigue Subscale Score, presence of severe fatigue, insomnia as PHQ-9 item 3), functional status (FACIT-Dyspnea Raw Functional Limitation Score, six-minute walk distance, need for assistance with daily activities), disease severity and organ damage (NAC stage, NT-proBNP, high-sensitivity troponin T, estimated glomerular filtration rate, time since diagnosis), congestion (peripheral oedema, jugular venous distension, hepatic venous congestion, tricuspid regurgitation peak velocity, E/e’), treatment (treatment status at the time of the survey, treatment class, treatment duration), and social situation (living alone, children, grandchildren, communication about worries). All univariable models were adjusted for age and sex. The multivariable models combined one indicator per domain in order to avoid collinearity between the overlapping symptom and function measures, and robustness was assessed by exchanging these indicators, as reported in the Results. No correction for multiple testing was applied, and the regression results are, therefore, hypothesis-generating.

**Sensitivity analyses.** Three prespecified sensitivity analyses were performed: exclusion of patients with a pre-existing anxiety disorder or depression that had begun before the manifestation of amyloidosis, or with chronic psychotropic medication; inclusion of treatment status, treatment class and treatment duration as covariates; and stratification by sex.

All analyses were performed using StataNow 18.5 Basic Edition (StataCorp, College Station, TX, USA).

## 3. Results

### 3.1. Cohort Derivation and Questionnaire Completion

The derivation of the patient sample is shown in Figure 1. Questionnaires were administered between July 2020 and February 2024. Of the 113 patients included in the analysis, 110 (97.3%) returned analyzable sets of questionnaires. Complete responses were available for the Distress Thermometer in 110 patients (100%), for the PHQ-9 in 109 (99.1%), for the GAD-7 in 108 (98.2%), for the FACIT-Fatigue in 107 (97.3%), for the SF-36 in 103 (93.6%), and for the PA-F-KF in 71 patients (64.5%). The PA-F-KF thus showed markedly higher item-level missingness than all other instruments. The gaps arose exclusively from the questions on occupational functioning and time off work, which are not relevant in a patient population consisting predominantly of pensioners. For the PA-F-KF, extrapolated sum scores were used accordingly, and a sensitivity analysis was added.

### 3.2. Characterization of the Patient Sample

The diagnosis was established histologically in 48 patients (42.5%), including 45 endomyocardial biopsies and 3 extracardiac biopsies (rectal mucosa in two patients, urinary bladder in one), and bone scintigraphy was positive in 84 patients (74.3%). Confirmation rested on histology alone in 29 patients (25.7%), on scintigraphy alone in 65 (57.5%) and on both modalities in 19 (16.8%). A concomitant monoclonal gammopathy occurred in 14 patients (12.3%). In all of them, the ATTRwt amyloidosis diagnosis was subsequently confirmed histologically with amyloid subtyping, thereby excluding AL amyloidosis. TTR genotyping was performed in 112 of 113 patients without evidence of an amyloidogenic TTR variant. In one patient, genotyping was not performed because reimbursement was declined in the absence of therapeutic consequences. Given the typical clinical presentation and the patient’s age, wild-type disease was assumed.

The mean age was 79.0 ± 5.9 years, and 14.2% of patients were female. 62% of patients were in good general condition with unrestricted age-appropriate performance status (ECOG 0). 27.8% of patients reported mild symptoms preventing physically demanding work (ECOG 1), and 10.2% had more severe limitations in daily activities (ECOG 2). The mean Charlson comorbidity index was 5.7 ± 1.6, and 64.6% of patients showed cardiac comorbidities. The average number of prescribed drugs was found to be 8.1 ± 3.1. Almost all patients had cardiac manifestations (99.1%), and nearly half were classified as NAC stage II-III at evaluation. NYHA functional class III was reported in 45.9% of patients, while 54% were categorized as NYHA functional class I-II.

Laboratory and echocardiographic findings are summarized in Supplementary Table S1. In brief, the mean NT-proBNP was 3107 ± 2950 pg/mL, mean high-sensitivity troponin 52.7 ± 34.0 pg/mL and mean estimated glomerular filtration rate 56.6 ± 17.4 mL/min. Echocardiography showed a mean interventricular septal thickness of 18.9 ± 3.1 mm, a mean posterior wall thickness of 15.1 ± 2.0 mm and a mean left ventricular ejection fraction of 52.7 ± 10.4%. The mean global longitudinal peak strain was −10.6 ± 3.6%. Clinical signs of congestion were present in a relevant proportion of patients. Half exhibited atrial fibrillation or flutter; a pacemaker rhythm was found in 15%. The average six-minute walk distance was 321 ± 122 m, with 11.3% of patients discontinuing the test prematurely.

Information regarding neurological involvement was available in 81 patients (71.7%). Among these, polyneuropathy was documented in 58 patients, corresponding to 71.6% of those. In a substantial proportion of these patients, competing metabolic or renal explanations were documented, and the etiology of the neuropathy remained ambiguous. Walking ability was preserved in the large majority (Coutinho stage I in 85.8%), and no patient was in Coutinho stage III. Connective tissue manifestations were frequent, with carpal tunnel syndrome in 67.0%, lumbar spinal stenosis in 28.4% and spontaneous tendon rupture in 6.6% of patients with available data.

On average, 21.2 + 19.3 months had passed since the initial diagnosis. Overall, 93.8% received disease-modifying treatment in general, and 78.8% were on causal treatment at the time of the questionnaire-based survey. Treatment was homogeneous; all 106 treated patients received the TTR stabilizer tafamidis. Of these, 81 received tafamidis as their sole disease-modifying treatment, while 25 were simultaneously enrolled in a randomized phase III trial of a gene-silencing agent and additionally received blinded study medication, so that allocation to a gene-silencing agent or placebo is unknown. No patient received an unblinded gene-silencing agent. Seven patients received no disease-modifying treatment, in two cases because of competing malignant disease. Treatment initiation preceded questionnaire completion in all treated patients, and the median treatment duration at the time of the survey was 12.1 months (mean 15.0 ± 12.1 months, n = 72 with a documented start date).

Regarding the social situation (Table 1), 77.5% of patients reported being in a partnership, 18.9% were widowed, and 3.6% were divorced. Almost all patients had children (97.3%), and most were grandparents (86.0%). The majority relied on an old-age pension (97.3%); 1.8% were still working or received occupational disability pension (0.9%). Care support was required by 28.2%, primarily at levels I-III (I 20.8%, II 41.7%, III 33.3%, IV 4.2%). A severe disability status had been assigned to 47.3%. Support with everyday activities was required by 42.7% of patients. Primary caregivers were usually partners (75.9%) or children or grandchildren (58.9%), whereas other relatives, friends, care services, etc. played a minor role. Participation in self-help groups was rare (4.5%).

### 3.3. Self-Assessment and Preexisting Psychological Disorders

Most patients reported impaired quality of life as a result of amyloidosis, which more than half rated as moderate or severe (Table 2). Around half of patients indicated increased anxiety since the onset of amyloidosis-related symptoms, mostly to a minor degree. Quality-of-life impairment due to anxiety affected 42.5% of patients and was mild in half of these patients. Quality-of-life impairment due to depression was more common, affecting 58% of patients, but was predominantly mild. A pre-existing psychological disorder that had begun before the manifestation of amyloidosis was documented in two patients, and five patients were on chronic psychotropic medication, comprising three anxiolytics and two antidepressants.

### 3.4. Questionnaire Results in the Total ATTRwt Amyloidosis Cohort

ATTRwt amyloidosis patients showed an average Raw Dyspnea Score of 11.7 ± 9.1 and a Raw Functional Limitation Score of 10.6 ± 8.4 on the FACIT-Dyspnea, corresponding to a Scale Score of 50.7 (Table 3). The average Fatigue Subscale Score was 33.0 + 12.0, with 35.8% of patients exhibiting severe fatigue (Table 3).

The Distress Thermometer yielded an average score of 5.1 ± 2.3 points, with 62.7% screening positive for distress. The mean GAD-7 sum score was 5.3 ± 4.5. According to the GAD-7, half of the patients showed no abnormalities, while 30.0%, 15.5% and 2.7% showed mild, moderate and severe anxiety symptoms, respectively, so 18.2% reached the threshold of ≥10 points. In about one-third of patients, anxiety had no impact on everyday life, while 44.4% reported mild and one in five patients reported relevant limitations in everyday life.

The PHQ-9 sum score averaged 5.9 ± 4.2 points. Moderate, moderately severe and severe depressive symptoms according to the sum score were found in 13.6%, 5.5% and 0% of patients, respectively, so19.1% reached the threshold of ≥10 points. According to the categorical evaluation of the PHQ-9 questionnaire, 10% of patients fulfilled the criteria for major depression and 10% those for other depressive syndromes. Taken together, 29 of 110 evaluable patients (26.4%) screened positive for anxiety and/or depression, as defined in Section 2.3. Of these, 8 met only the GAD-7 criterion, 9 only the PHQ-9 criterion and 12 both criteria.

Because two items were not applicable to most of the cohort, three scoring approaches were compared in the 109 patients who completed at least ten of the twelve PA-F-KF items. The extrapolated 12-item score used for the primary analysis yielded a mean of 25.69 ± 8.65, with 18 patients (16.5%) above the cut-off. A sum score restricted to the ten universally applicable items and rescaled to the 12-item metric yielded 26.87 ± 8.96 with 22 patients (20.2%), and an instrument-conform score assigning the lowest value to non-applicable items yielded 24.79 ± 7.84 with 15 patients (13.8%). Classification agreed in 93.6% to 97.2% of patients across the three approaches, and the scores correlated closely (r ≥ 0.987). The prevalence estimate is, therefore, robust to the handling of non-applicable items.

### 3.5. Comparison with the Age-Matched General Population

**Quality of life compared with the general population.** Based on the SF-36 results, both summary scales were significantly lower than in the age-matched general population, and to a comparable degree. The Physical Component Summary was 36.9 ± 9.8 versus 43.5 ± 11.8 (*p* < 0.001), corresponding to a medium effect size (Cohen’s d = −0.59), and the Mental Component Summary was 46.8 ± 9.1 versus 52.2 ± 10.3 (*p* < 0.001, Cohen’s d = −0.54). Both differences exceed the minimal clinically important difference of approximately three to five points for the SF-36. All subscales showed significantly lower scores (*p* < 0.001), except for the Bodily pain subscale, compared to the age-matched norm group (Table 4). The largest deficits were observed for General Health (44.4 vs. 62.0, *p* < 0.001) and Vitality (46.1 vs. 61.5, *p* < 0.001). That is, in the domains most closely related to physical energy and perceived health.

### 3.6. Patient Characteristics Depending on Positive Screening Results for Anxiety (GAD-7) and/or Depression (PHQ-9)

**Patients screening positive for anxiety and/or depression.** Twenty-nine of 110 evaluable patients (26.4%) screened positive for anxiety and/or depression, defined as a GAD-7 sum score ≥ 10 and/or a PHQ-9 sum score ≥ 10 (Section 2.3). When comparing patient characteristics of those with positive screening results for anxiety and depression, no significant differences were found regarding age, sex, BMI, or comorbidities. Significant differences exist in ECOG, NYHA functional class, congestion (using jugular distention and E/e’ as surrogate), cardiac biomarkers, renal function, physical performance (6 min walking test), need for assistance, and severe disability (Table 1).

Patients positively screened for anxiety and/or depression achieved significantly higher Raw Dyspnea (18.5 ± 8.6 vs. 9.2 ± 8.0, *p* < 0.001) and Raw Functional limitation scores (16.6 ± 8.3 vs. 8.4 ± 7.4, *p* < 0.001) corresponding to Scale Scores of 27.7 vs. 47.8 and 61.9 vs. 48.3, respectively. At the same time, they more frequently showed severe fatigue (75.9% vs. 21.3%, *p* < 0.001) and a poorer mean Fatigue Subscale Score (20.8 ± 9.0 vs. 37.5 ± 9.7, *p* < 0.001). General quality of life measured by the SF-36 showed significantly worse results across all scales in patients with anxiety and/or depression (*p* < 0.01 for all scales including the mental component summary). When comparing the quality of life of patients with ATTR amyloidosis and a PHQ-9 sum score >10 with the age-matched norm group with abnormal PHQ-9 results published by Ellert et al. (Table 4), ATTRwt amyloidosis patients showed a comparable mental component summary (38.1 ± 6.7 vs. 39.5 ± 10.3, *p* = 0.61) and a comparable physical component summary (31.1 ± 8.3 vs. 31.9 ± 12.8, *p* = 0.83). General Health was significantly worse (27.2 ± 14.0 vs. 38.3 ± 16.9, *p* < 0.05). All other subscales showed non-significant differences but a trend toward worse quality of life, except for Physical Functioning and Bodily Pain.

**Distress and screening-positive anxiety or depression are not interchangeable.** Of the 69 patients with a positive distress screening, 41 (59.4%) screened positive for neither anxiety nor depression. Conversely, only one patient with a positive anxiety or depression screen was distress-negative. The Distress Thermometer, therefore, identified a considerably broader group, the majority of which does not meet the thresholds usually taken to indicate a need for further psychiatric assessment.

### 3.7. Predictors of Reduced Quality of Life, Distress and Mental Health Conditions

Univariable and multivariable regression analyses adjusted for age and sex are reported in Supplementary Table S2. Predictors were clustered into three domains. First, the indicators of general condition, symptom burden and functional limitation predicted all six outcomes consistently. ECOG performance status, dyspnea (NYHA functional class or Raw Dyspnea Score), functional limitation (Raw Functional Limitation Score and six-minute walking distance), fatigue (Fatigue Subscale Score and severe fatigue), insomnia (PHQ-9 item 3) and need for assistance in daily life were significantly associated with distress, anxiety, depression, fear of progression and with both summary scales of the SF-36. Second, the markers of cardiac disease severity and organ damage showed a more restricted and outcome-specific pattern. High-sensitivity troponin T was the most consistent of these, being associated with distress, anxiety, depression, fear of progression and the mental component summary, but not with the physical component summary. NT-proBNP was associated with distress only, and renal function (eGFR) with distress, depression and the physical component summary. NAC stage at the time of the survey was associated with the physical component summary only. Among the congestion surrogates, E/e’ was associated with depression and jugular venous congestion with the mental component summary, and peripheral edema, hepatic venous congestion, and tr-v_max_ showed no association with any outcome. Notably, the markers of amyloid load and cardiac remodeling such as wall thickness, left ventricular ejection fraction and global longitudinal peak strain showed no association with any psychological outcome. Time since disease onset was associated with depression only and showed no association with distress, anxiety, fear of progression or either summary scale. Third, the social variables were largely without influence; only the presence of children was associated with the physical component summary.

When combining various predictors in multivariate regression models (Table 5), severe fatigue was the only independent predictor of the mental component summary and was robust across both model variants (b = −7.97 and −8.06, both *p* < 0.001). The physical component summary was predicted by dyspnea, whether operationalized as the Raw Dyspnea Score (b = −0.36, *p* = 0.015) or as NYHA functional class (b = −4.44, *p* = 0.007), and by female sex in both variants (b = −4.98 and −5.04, both *p* < 0.05). Insomnia predicted the physical component summary in the second model variant only (b = −3.26, *p* = 0.001). Distress was independently predicted by ECOG performance status, dyspnea, and, inversely, by age (b = −0.14 and −0.16, both *p* < 0.001), while severe fatigue and insomnia predicted both anxiety and depression across both variants.

In sex-stratified models adjusted for age (Supplementary Table S3), the predictor structure was concordant across sexes. Fatigue significantly predicted the GAD-7, PHQ-9 and Distress Thermometer in both men (b = −0.10 to −0.25, all *p* < 0.001) and women (b = −0.11 to −0.38, *p* ≤ 0.046), as did ECOG performance status (men b = +1.49 to +2.19, all *p* ≤ 0.005; women b = +1.62 to +5.37, *p* = 0.001 to 0.074). Point estimates were larger in women, but confidence intervals overlapped throughout. So, with only 16 women, this is not interpreted as a sex difference.

**Disease stage and functional disability** (Supplementary Table S4)**.** Comparing NAC stage II–III with stage I in models adjusted for age and sex, no association reached significance for any outcome: b = +1.02 for the GAD-7 sum score (*p* = 0.261), +0.64 for the PHQ-9 sum score (*p* = 0.452), +0.59 for the Distress Thermometer (*p* = 0.202), +2.22 for the PA-F-KF sum score (*p* = 0.207), −3.55 for the physical component summary (*p* = 0.068) and −1.57 for the mental component summary (*p* = 0.399). In contrast, walking ability showed a consistent pattern. Coutinho stage II, that is, the need for a walking aid, was compared with stage I in the same models. Stage II was associated with a higher PHQ-9 sum score (b = +2.60, 95% CI 0.14 to 5.05, *p* = 0.038) and a lower physical component summary (b = −5.83, 95% CI −11.49 to −0.16, *p* = 0.044), with non-significant effects in the same direction for anxiety (b = +1.74, *p* = 0.198), distress (b = +0.71, *p* = 0.303), fear of progression (b = +4.07, *p* = 0.119) and the mental component summary (b = −3.47, *p* = 0.209). Given that only 14 patients were in stage II, these results should be regarded as hypothesis-generating. They nevertheless indicate that gait disability and the associated loss of independence are accompanied by more pronounced depressive symptoms and greater physical limitation, but not by higher levels of nonspecific distress.

**Treatment** (Supplementary Table S5)**.** Seven patients (6.2%) received no disease-modifying treatment. All treated patients were given the TTR stabilizer tafamidis; 25 of them (22.1% of the cohort) were simultaneously enrolled in a phase III trial of a gene-silencing agent and additionally received blinded study medication, so that no patient was exposed to a gene-silencing agent outside a trial and allocation to verum or placebo is unknown. In models adjusted for age and sex, neither treatment status (all *p* ≥ 0.18) nor treatment at the time of the survey (all *p* ≥ 0.22), nor treatment class, was associated with distress, anxiety, depression, fear of progression, fatigue or either summary scale. For the treatment class, all coefficients relative to untreated patients were small and non-significant (all *p* ≥ 0.16). Treatment duration, available for 72 patients, was associated with a lower physical component summary (b = −2.39 per year, 95% CI −4.37 to −0.41, *p* = 0.019) and showed a trend towards higher distress (b = +0.49, *p* = 0.058), with no association for the remaining outcomes (all *p* ≥ 0.20). Since longer treatment duration also reflects a longer disease course, this finding is more plausibly interpreted as an effect of disease duration than of treatment itself.

**Sensitivity analysis excluding pre-existing psychiatric disorders.** Repeating all analyses after exclusion of the five patients with a pre-existing anxiety disorder or depression or with chronic psychotropic medication left the results essentially unchanged (Supplementary Table S6). The proportion screening positive for anxiety and/or depression was 25.7% instead of 26.4%. The proportion with a GAD-7 ≥ 10 was 17.1% instead of 18.2%, and the proportion with a PHQ-9 ≥ 10 was 19.0% instead of 19.1%. The mean scores likewise changed only marginally, and the multivariable models were unchanged in sign and significance.

**Contribution of ATTR-specific features beyond heart failure severity.** In hierarchical regression models (Supplementary Table S7), ATTR-specific features (NAC stage, neurological involvement, Coutinho stage, carpal tunnel syndrome) were added to a base model comprising markers of heart failure severity (ECOG, log NT-proBNP, LVEF, six-minute walk distance, age, sex). The ATTR-specific features added no significant explanatory value to any of the four outcomes: ΔR^2^ = 0.051 for anxiety, 0.096 for depression, 0.060 for distress and 0.069 for the FACIT-Fatigue Fatigue Subscale Score (all *p* ≥ 0.15, n = 65). Psychological burden in this cohort is therefore not explained by amyloid-specific disease features beyond the severity of heart failure. Consistent with this, patients in the NAC stage I group (n = 70) showed essentially unchanged prevalences compared with the full cohort: 17.1% versus 18.2% for a GAD-7 ≥ 10, 17.1% versus 19.1% for a PHQ-9 ≥ 10, 61.4% versus 62.7% for a positive distress screen and 34.3% versus 35.8% for severe fatigue.

### 3.8. Coping Strategies

Approximately half of patients with positive screening results talked about their anxiety, while around a quarter of patients would like to discuss it but did not. In multivariate linear regressions adjusted for age and sex (Table S9), physical activity showed a significant association with lower levels of distress, anxiety, depression, and fear of progression, and with higher PCS scores, while no significant influence on MCS was observed. Cultural activity was associated with significantly lower levels of depression and fear of progression. Because of the cross-sectional design, these associations must not be interpreted causally. It cannot be determined whether coping activity reduces psychological burden or whether lower burden and better physical function enable such activity.

If surrogate parameters for dyspnea and functional limitation (Model 1: Raw Dyspnea Score, Raw Functional Limitation Score; Model 2: NYHA functional class and 6 min walking distance), as well as fatigue (presence of severe fatigue), were included, physical activity remained a significant predictor of lower anxiety (all covariates not significant) and depression (fatigue also significant), while PCS was primarily determined by sex and shortness of breath (tables available upon request).

## 4. Discussion

### 4.1. Main Findings

Cardiac ATTR amyloidosis represents an increasingly frequently diagnosed cause of heart failure and has recently become treatable since the approval of tafamidis in 2020, with additional new treatment options steadily emerging [1,2,5]. Similar to heart failure in general, where depression has a known prevalence of 20–40% [10], psychological distress is not uncommon among patients with amyloidosis [15,16,17]. However, available data on its prevalence and potential predictors in ATTR amyloidosis are neither comprehensive nor consistent.

In the light of this, we characterized the psycho-emotional profile and psychological disease burden in patients with systemic wild-type ATTR amyloidosis in detail using a multidimensional set of questionnaires assessing general (health-related) quality of life, distress, anxiety, depression, and fear of progression, as well as symptom burden (dyspnea, fatigue), within the clinically well-phenotyped ATTRwt amyloidosis patients of the AmyKoS cohort study.

The key findings were the following:

- Both physical and mental health-related quality of life were markedly impaired with a medium effect size and exceeding the minimal clinically important difference;
- Overall, 88.4% of patients rated their quality of life as impaired, and half experienced increased anxiety since diagnosis of amyloidosis;
- Nonspecific distress was common, affecting approximately two-thirds of patients. Importantly, almost 60% of distress-positive patients screened positive for neither anxiety nor depression;
- Mild anxiety and depressive symptoms occurred frequently, while approximately one quarter of patients (26.4%) screened above the thresholds usually taken to indicate a need for further clinical assessment (GAD-7 or PHQ-9 ≥ 10 points);
- About one-third of patients were affected by severe fatigue;
- 9.2% screened positive for fear of progression, which is low compared with oncological cohorts;
- Patients with signs of anxiety and/or depression showed worse results regarding ECOG, symptom burden including dyspnea and fatigue, NYHA functional class, congestion, cardiac biomarkers and renal function, physical performance, need for assistance, and severe disability;
- Predictors for impaired quality of life and mental health include general condition, symptom burden (dyspnea, fatigue, insomnia), and functional limitation/need for assistance, whereas markers of amyloid load such as wall thickness, ejection fraction and longitudinal strain showed no association;
- Gait disability (Coutinho stage II) was associated with more depressive symptoms and a lower physical component summary, but not with higher nonspecific distress;
- Active coping strategies such as physical and creative activities were associated with a lower prevalence of mental health symptoms, although causality cannot be inferred.

In this ATTRwt cohort, both dimensions of health-related quality of life are substantially impaired. Nonspecific distress is frequent and appears to track symptom burden and loss of function rather than psychiatric morbidity in the narrower sense, since almost 60% of distress-positive patients screen positive for neither anxiety nor depression. A smaller subgroup of approximately one-quarter of patients shows anxiety or depressive symptoms at a level that warrants further clinical evaluation.

Our SF-36 results can be compared with those recently reported by Sanchorawala et al. in a large North American referral cohort [8], since both studies used norm-based scoring against the US general population. In their ATTRwt subgroup of 407 patients, the physical component summary was 36 ± 11 and the mental component summary 51 ± 11, compared with 36.9 ± 9.8 and 46.8 ± 9.1 in our cohort. The physical component summary thus replicates their finding almost exactly, whereas our mental component summary is approximately four points lower. A direct comparison of disease stage is not possible, since Sanchorawala et al. did not report standardized measures of cardiac severity such as NAC stage, natriuretic peptides or ejection fraction, and explicitly acknowledge this as a limitation. Indirect indicators nevertheless argue against more advanced disease in our cohort. Their observation period spans 1992 to 2022 and thus largely predates both non-invasive diagnosis and disease-modifying treatment, and the authors themselves attribute part of the functional impairment they observed to delayed recognition after a prolonged diagnostic journey. In our cohort, 63.7% were in NAC stage I, the median NT-proBNP was 2456 pg/mL, and 93.8% received disease-modifying drugs. The near-identical physical component summary in both cohorts is consistent with a comparable degree of functional impairment. A more plausible explanation for the difference in the mental component summary is the higher questionnaire completion rate in our study (97.3% versus 83.9%), which reduces the selective loss of more severely affected and more distressed patients, together with the differences between the German and US versions of the instrument.

The demographic profile of our cohort also corresponds to the established ATTRwt phenotype. In the THAOS registry, 49.1% of 1251 patients with ATTRwt amyloidosis were aged 70 to 79 years and 34.5% were aged 80 to 89 years, which matches our mean age of 78.95 years. Men accounted for more than 90% of that registry cohort, with the proportion of women rising with age from 4.1% in patients under 70 to 14.3% in those aged 90 or above, so that our 14.2% of women is at the upper end of, rather than below, the expected range [31]. Taken together with the near-identical SF-36 values observed in an independent healthcare system, this suggests that the core findings are not center-specific.

### 4.2. Potential Mechanisms

The pattern of predictors observed here suggests that the psychological burden in ATTRwt amyloidosis is mediated primarily by functional and psychosocial consequences of the disease rather than by amyloid deposition as such. Need for assistance with daily activities was the strongest single discriminator between patients with and without a positive screening result (82.1% versus 29.1%), and general condition, dyspnea, fatigue, insomnia and walking capacity were consistent predictors, whereas wall thickness, ejection fraction and global longitudinal peak strain were not. The newly observed association of Coutinho stage II with depressive symptoms and fatigue fits this interpretation, since loss of ambulation directly restricts social participation and independence.

Fatigue deserves particular attention, and it should be understood as a multidimensional symptom rather than as a specific manifestation of amyloidosis. In this elderly cohort, plausible contributors include cardiac output limitation and congestion, chronotropic incompetence given the high prevalence of conduction disease and pacemaker rhythms, autonomic and peripheral neuropathy, renal dysfunction, physical deconditioning, sleep disturbance, depression and anxiety, and polypharmacy. Insomnia was frequent. In models adjusted for NYHA class, renal function, medication count, insomnia and pulmonary and endocrine comorbidity, fatigue remained associated with depressive symptoms and with the physical component summary, indicating that it is not merely a proxy for cardiac severity. We nevertheless cannot exclude that anemia, iron deficiency, thyroid dysfunction or sleep-disordered breathing contribute substantially, since these were not systematically assessed.

The number of prescribed drugs was significantly higher in patients screening positive for anxiety or depression (9.2 versus 7.8). While several drug classes used in this population can contribute to fatigue, sleep disturbance or depressive symptoms, the more parsimonious interpretation is that medication count reflects overall disease severity and comorbidity burden rather than exerting a direct pharmacological effect on mood.

A question that follows directly from the high prevalence of cardiac involvement is how far the burden observed here is specific to ATTR amyloidosis rather than shared with chronic heart failure in general. A definitive separation would require a concurrently assessed heart failure control group, which was not available. Three observations nevertheless allow for a differentiated answer. First, ATTR amyloidosis-specific features added no significant explanatory value beyond heart failure severity for anxiety, depression or distress, whereas they did so for fatigue. Second, patients in NAC stage I showed prevalences essentially identical to the full cohort, indicating that the burden is not driven by cardiologically advanced disease. Third, the prevalences of anxiety and depression observed here lie within the range reported for heart failure cohorts [32,33]. Taken together, psychological burden in the narrower sense appears largely not to be ATTR amyloidosis-specific but rather an expression of chronic symptomatic cardiac disease in advanced age, whereas fatigue shows a component beyond heart failure severity that is associated with ATTR amyloidosis-typical features.

Finally, a possible contribution of cerebral amyloid deposition to behavioral and affective symptoms seems unlikely, because leptomeningeal and cerebral amyloid involvement is described for specific ATTRv variants but is not established for the wild-type form, and our cohort contained no patient with an amyloidogenic variant.

The findings are not surprising and are consistent with previous publications. Fatigue is one of the most common initial symptoms of light chain (AL) amyloidosis, with a prevalence of 60–70% [34]. Evidence regarding fatigue in ATTR amyloidosis is scarce. According to the recent review by Cook et al. 2026, the prevalence at initial diagnosis has been estimated to be <20% [34]. However, to the authors’ knowledge, there are no data on fatigue throughout the course of the disease. In this study, we were able to demonstrate for the first time that fatigue is widespread among patients with ATTRwt amyloidosis and is one of the most important predictors of reduced quality of life and anxiety or depression. Consistent with this, patients with cardiac and neuropathic manifestations indicated in focus groups that fatigue is one of the most challenging symptoms [9]. When comparing the severity of fatigue with the published results from the FACIT-Fatigue questionnaire in cancer patients, the severity of fatigue in ATTRwt amyloidosis appears at least comparable to that in cancer or stroke patients [35]. This comparison must, however, be interpreted with considerable caution, since the populations differ substantially in age, comorbidity and treatment context, and since the FACIT-Fatigue was administered at different points in the disease trajectory. It indicates an order of magnitude rather than an equivalence. Fatigue is also a known accompanying symptom in heart failure and is, therefore, not specific to amyloidosis [36]. The literature suggests that higher levels of fatigue correlate with poorer cardiac function [36], which is consistent with observations in our ATTRwt amyloidosis cohort using the NAC stage as a surrogate parameter. The observed relationship between fatigue and anxiety or depression is not new [37].

The prevalence of anxiety and depressive symptoms found in our cohort is consistent with that reported by Smorti et al. 2023 [15]. Similarly, the prevalence of depression is comparable to the findings of Eldhagen et al. 2023 [16]. Consistent with this, a prevalence of anxiety and depression in up to half of patients is reported in heart failure cohorts [32,33], but also in the general population [38]. At first glance, the low prevalence of previously diagnosed anxiety disorders and depression (<4%) appears surprising given the known prevalence of depressive and anxiety symptoms of 21.9% and 14.3%, respectively [38]. This may be explained as a generational phenomenon. Looking at the cohort of 75- to 79-year-olds within the DEGS1 dataset, the lifetime prevalence of depression was 8.7% and that of anxiety disorders 3.6% [30]. Moreover, Walther et al. 2025 found a lower prevalence for depressive (12.2%) and anxiety symptoms (6.7%) in the elderly [38]. Our observation that anxiety and depression are associated with poor general condition, symptom burden and need for assistance is consistent with the findings of Ponti et al. [17] and the results of Rintell et al., who identified in focus groups that activity, inability to exercise, insomnia, and fatigue are the most burdensome symptoms [9].

Furthermore, we were able to quantify ‘fear of progression’ in an ATTRwt amyloidosis cohort for the first time. Depending on the operationalization, 9.2% of patients met the item-based criterion according to Hefner et al. and 16.5% exceeded the sum score cut-off of 34 points. Both estimates are low compared to cancer cohorts such as those with colorectal carcinoma, which show a prevalence of 36–50% [29,39]. One plausible explanation specific to the wild-type form is that concerns about heritability, genetic counseling and family screening, which contribute substantially to disease-related fear in ATTRv amyloidosis and in hereditary cancer syndromes, are largely absent in this population. A further consideration is that the PA-F-KF was developed in oncological settings and may not capture the concerns most relevant to elderly patients with a chronic cardiac condition, such as progressive breathlessness, loss of mobility and caregiver dependence. Both considerations argue for the development and validation of disease-specific instruments. Comparative data from European heart failure cohorts are scarce. The only closely comparable European study reports markedly higher fear of progression in patients with a left ventricular assist device [40], which is plausible given the far greater treatment burden and the younger age of that population. An Asian heart failure cohort reports a prevalence of 34.8% [41], but differences in health care context and in the instruments used limit direct comparison. Taken together, fear of progression appears to be less pronounced in ATTRwt amyloidosis than in the chronic conditions studied so far.

### 4.3. Implications for Clinical Assessment and Management

Three considerations follow from these findings. First, all results reported here are based on self-report screening instruments. A positive screening result indicates the need for further psychological or psychiatric assessment to establish a diagnosis; it does not constitute a diagnosis in itself. This distinction is essential, given that almost 60% of distress-positive patients screened positive for neither anxiety nor depression.

Second, the predictors identified here point to the drivers of burden and thereby to the most promising points of intervention. Since general condition, symptom burden, functional limitation and need for assistance were consistently associated with psychological outcomes, whereas markers of amyloid load were not, optimization of heart failure treatment and volume status, treatment of insomnia, structured exercise therapy and rehabilitation, and social work support with care level applications and assistance in daily life address the burden at its source for a substantial proportion of patients.

Third, pharmacological treatment will nevertheless be required for some patients, and here, particular caution is warranted. Conduction disease is frequent. Fifteen percent of our patients had a pacemaker rhythm, and the mean number of prescribed drugs was 8.1. So, QTc prolongation, orthostatic hypotension in the presence of autonomic neuropathy, fall and delirium risk, and drug interactions all require attention. Treatment decisions should be made individually and in consultation with psychiatry or clinical pharmacology rather than following a generic algorithm. A recent review provides a comprehensive overview of symptomatic management in transthyretin amyloidosis, including autonomic dysfunction and its systemic consequences [42].

### 4.4. Clinical Implications and Future Directions

Our findings support the integration of a stepwise screening approach into routine amyloidosis care. The Distress Thermometer is a single-item instrument that can be completed in seconds and identifies a broad group of burdened patients. Because almost 60% of distress-positive patients in our cohort screened positive for neither anxiety nor depression, a positive distress screen should not automatically trigger referral for psychiatric assessment. Instead, it should prompt a structured conversation about what is driving the burden, followed by targeted second-step screening with the GAD-7 and PHQ-9, where emotional symptoms predominate.

In our cohort, the conventional Distress Thermometer cut-off of five points identified a positive anxiety or depression screen with a sensitivity of 96.6% and a negative predictive value of 97.6%, so that a second step could be omitted in 37.3% of patients. Specificity was 49.4% and the positive predictive value 40.6%, which is precisely why a second step is required rather than direct referral. A cut-off of six points would be more efficient (sensitivity 89.7%, specificity 72.8%, second step needed in 43.6% of patients) at the cost of missing three patients. Because both instruments were administered simultaneously in the same cohort, these figures describe agreement rather than constituting an independent validation.

This distinction has direct consequences for care. For a substantial proportion of patients, the appropriate response is the optimization of heart failure treatment and volume status, treatment of insomnia, structured physical rehabilitation and exercise training, assistance with daily activities, and social work support for care level applications and disability certification. These measures address precisely the predictors that emerged consistently in our analyses. For the smaller group with clinically relevant anxiety or depressive symptoms, access to psycho-oncological or psycho-cardiological support and, where indicated, pharmacological treatment as outlined above should be available within the multidisciplinary amyloidosis clinic rather than requiring external referral.

Operationally, we suggest allocating patients with a positive distress screen according to the dominant driver of burden identified in the second step: optimization of heart failure treatment, volume status and insomnia where symptom burden predominates; structured exercise therapy and cardiac rehabilitation where functional limitation predominates; and social work support with care level applications, disability certification and help with daily activities where need for assistance predominates. To meet these complex needs, we require center structures with a multi-professional team.

Several questions remain open. Longitudinal data are needed to determine whether the psychological burden changes with disease progression and with treatment, particularly now that survival has improved substantially. The association between coping activity and lower symptom levels observed here is cross-sectional and requires prospective evaluation, ideally in the form of intervention studies of structured exercise or activity programs. Finally, the absence of ATTR amyloidosis-specific instruments for distress and fear of progression limits the interpretation of these constructs, and the development of disease-specific patient-reported outcome measures, which are currently underway, should be extended to the psychological domain.

### 4.5. Strengths

This study has several strengths. First, the cohort was homogeneous, so that the findings apply unambiguously to wild-type ATTR amyloidosis rather than to a mixed ATTR population. Second, the psychological assessment is multidimensional. Five validated instruments covering quality of life, distress, anxiety, depression and fear of progression were administered concurrently, which made it possible to separate nonspecific distress from anxiety and depressive symptoms at clinically meaningful thresholds, a distinction that single-instrument studies cannot make. The additional structured assessment of social aspects and coping strategies provided relevant supplementary information, which facilitates the interpretation of the data. To our knowledge, fear of progression has not previously been quantified in ATTR amyloidosis. Third, the psychological data are linked to a detailed clinical characterization within a prospective cohort study, including standardized echocardiography, cardiac biomarkers, six-minute walk testing, disease staging and complete treatment documentation, which allowed the psychological findings to be related to objective measures of disease severity and functional capacity rather than to self-report alone. Fourth, the response rate was high, with 110 of 113 patients returning evaluable questionnaires, and the comparison with an age-matched sample from a national health survey provides an external reference that is rarely available in rare-disease cohorts. Finally, the close agreement of our SF-36 results with those from an independent North American referral cohort supports the external validity of the core findings.

### 4.6. Limitations

Several limitations should be considered. The study was conducted at a single specialized referral center, which entails both referral bias and a case mix that may not represent patients managed in general cardiology practice. The design is cross-sectional, so no causal inferences can be drawn.

The reasons for non-inclusion of the remaining 21 patients of the AmyKoS cohort were not systematically documented, so a selection bias cannot be excluded at all.

The cohort was predominantly male (85.8%) and very elderly (mean age 79.5 years) and consisted exclusively of patients with wild-type disease. The findings, therefore, cannot be generalized to ATTRv amyloidosis, to younger patients, to women, or to patients with advanced neuropathic disease, in whom loss of independence, heritability concerns and family screening are likely to shape the psychological burden differently. 

Since women in the general population report higher anxiety and depression scores, the predominance of men is expected to lead to an underestimation rather than an overestimation of psychological burden, so the comparison with the general population is conservative in this respect. With only 16 women, however, the power to detect sex differences was limited. Within the cohort, no significant sex differences were observed for the GAD-7 (5.15 ± 4.45 in men versus 5.88 ± 4.51 in women, *p* = 0.56), the PHQ-9 (5.76 ± 4.02 versus 6.44 ± 4.95, *p* = 0.61), the Distress Thermometer (*p* = 0.78) or the FACIT-Fatigue Fatigue Subscale Score (*p* = 0.71), and sex-stratified models showed a concordant predictor structure (Supplementary Table S3).

Generalizability is further limited by the healthcare context. Statutory long-term care insurance and formal disability status shape the findings on care level, disability certification and need for assistance, which are, therefore, not directly transferable to systems with different entitlements.

Neither the Distress Thermometer nor the PA-F-KF has been validated in ATTR amyloidosis or in elderly patients with chronic heart failure, and the cut-offs used derive from oncological populations. The PA-F-KF additionally showed the highest item-level missingness of all instruments, and extrapolated sum scores were used. So, the findings relating to fear of progression are exploratory. Several PHQ-9 items address sleep, energy, appetite and psychomotor changes, which have direct somatic explanations in a population with heart failure, fatigue and polypharmacy, so depressive symptoms may be systematically overestimated by the sum score. No structured clinical interview was performed, so the screening results cannot be equated with clinical diagnoses. Since the completion of this study, an ATTR amyloidosis-specific quality of life instrument, including an emotional wellbeing scale, has been developed and validated [12,13], which should facilitate more precise assessment of these constructs in future work.

Several clinical variables that would have permitted a more complete differential assessment of fatigue were not captured, e.g., iron status and thyroid function. Validated neurological disability measures beyond the Coutinho stage, such as the polyneuropathy disability score, the Medical Research Council sum score or the Neuropathy Impairment Score, were not assessed across the total cohort, and dedicated neurological assessment was performed on clinical indication rather than in all patients. So, the prevalence of neurological involvement refers to the assessed subgroup. Diagnostic latency, previous misdiagnoses and the course of the diagnostic pathway were not included in the analysis, although these may plausibly be more relevant to anxiety than time since formal diagnosis alone.

Finally, the predictor analyses were exploratory in nature. Since the clinical and questionnaire-based predictors overlap substantially in content and were examined in parallel models without correction for multiple testing, the regression results should be regarded as hypothesis-generating. Residual confounding by comorbidity, treatment and socioeconomic factors cannot be excluded.

Because the design is cross-sectional, the direction of the associations between symptom burden, functional status and psychological outcomes cannot be established, and reverse causation cannot be excluded. Because 93.8% of patients received disease-modifying treatment, the untreated comparison group was very small, and the treatment models correspondingly underpowered. Finally, the absence of a concurrently assessed heart failure control group means that the separation of ATTR-specific from generic heart failure burden remains partial.

Notwithstanding these limitations, this study provides an in-depth characterization of quality of life and psychological burden in a clinically well-phenotyped and homogeneous ATTRwt cohort, combining validated instruments across five psychological domains with a detailed social, clinical and functional assessment.

## 5. Conclusions

In patients with wild-type transthyretin amyloidosis, both physical and mental health-related quality of life are substantially impaired relative to the age-matched general population, and to a comparable degree. Self-reported impairment of quality of life affects approximately 90% of patients, and half report increased anxiety since diagnosis. Nonspecific distress is common, affecting about two-thirds of patients, but the majority of distress-positive patients do not screen positive for anxiety or depression. Approximately one quarter of patients screen above thresholds indicating a need for further clinical assessment, and fear of progression affects about one in ten. Predictors of impairment are general condition, symptom burden and functional limitation or need for assistance, with fatigue being widespread and central, whereas markers of amyloid load show no association. These findings argue for a stepwise screening approach and for a differentiated response, in which many burdened patients are best served by symptom control, rehabilitation and support with daily activities, while a smaller subgroup requires access to psychological or psychiatric care.

## Figures and Tables

**Table 1:** Characterization of the patient sample.

	Total Cohort				Anxiety and/or Depression						
					Yes			No			
	Mean		SD	n	Mean		SD	Mean		SD	*p*
age	78.95	±	5.90	113	78.36	±	6.15	79.00	±	5.55	n.s.
sex [% female]	14.2%			113	20.7%			12.3%			n.s.
BMI [kg/m^2^]	27.69	±	3.70	89	27.88	±	4.22	27.65	±	3.61	n.s.
ECOG 0	62.0%			108	37.9%			72.4%			**
1	27.8%			108	41.4%			21.1%			*p* < 0.1
2	10.2%			108	20.7%			6.6%			*p* < 0.1
3	0.0%			108	0.0%			0.0%			
NYHA functional class	2.37	±	0.63	113	2.66	±	0.57	2.27	±	0.61	**
I	7.1%			113	3.7%			7.4%			n.s.
II	46.9%			113	22.2%			58.8%			***
III	45.9%			113	74.1%			33.8%			***
IV	0.0%			113	0.0%			0.0%			
Charlson comorbidity index (score)	5.70	±	1.61	113	5.69	±	1.78	5.68	±	1.51	n.s.
cardiac comorbidities	64.6%			113	75.9%			61.7%			n.s.
number of drugs	8.13	±	3.05	113	9.21	±	3.05	7.83	±	2.99	*
organ involvement											
heart	99.1%			113	96.6%			100.0%			n.s.
polyneuropathy ^#^	71.6%			81	61.9%			75.4%			n.s.
other	1.8%			113	3.4%			1.2%			n.s.
NAC stage (PD) I	63.7%			113	55.2%			66.7%			n.s.
II	23.0%			113	37.9%			18.5%			*p* < 0.1
III	13.3%			113	6.9%			14.8%			n.s.
NAC stage (survey) I	56.6%			113	44.8%			60.5%			n.s.
II	32.7%			113	44.8%			29.6%			n.s.
III	10.6%			113	10.3%			9.9%			n.s.
Coutinho stage I	85.8%			113	79.3%			88.9%			n.s.
II	14.2%			113	20.7%			11.1%			n.s.
III	0.0%			113	0.0%			0.0%			
disease duration (months)	21.20	±	19.26	112	27.52	±	24.05	19.24	±	17.08	*p* < 0.1
specific treatment for ATTR amyloidosis											
in general	93.8%			113	93.1%			95.1%			n.s.
survey	78.8%			113	79.3%			82.7%			n.s.
marital status											
partnership	77.5%			111	75.9%			79.7%			n.s.
widowed	18.9%			111	20.7%			16.5%			n.s.
divorced	3.6%			111	3.4%			3.8%			n.s.
children	97.3%			110	96.4%			97.5%			n.s.
grandchildren	86.0%			107	89.3%			84.2%			n.s.
employment status											
employment	1.8%			110	0.0%			1.3%			n.s.
old-age pension	97.3%			110	96.4%			98.7%			n.s.
occupational disability pension	0.9%			110	3.6%			0.0%			n.s.
sick leave	0.0%			110	0.0%			0.0%			
care level	28.2%			110	39.3%			24.1%			n.s.
1	20.8%			24	20.0%			23.1%			n.s.
2	41.7%			24	30.0%			46.2%			n.s.
3	33.3%			24	40.0%			30.8%			n.s.
4	4.2%			24	10.0%			0.0%			n.s.
5	0.0%			24	0.0%			0.0%			
severe disability	47.3%			110	67.9%			39.2%			**
degree of severe disability	69.30	±	19.11	50	71.39	±	19.84	68.00	±	18.83	n.s.
need for assistance (everyday activities)	42.7%			110	82.1%			29.1%			***
primary caregiver	98.2%			112	100.0%			97.5%			n.s.
partner	75.9%			112	75.9%			77.5%			n.s.
children/grandchildren	58.9%			112	51.7%			61.3%			n.s.
other relatives	3.6%			112	10.3%			1.3%			n.s.
friends	3.6%			112	0.0%			5.0%			*
nursing service	5.4%			112	10.3%			3.8%			n.s.
domestic help	4.5%			112	6.9%			3.8%			n.s.
other	0.9%			112	0.0%			1.3%			n.s.
participation in self-help group	4.5%			112	6.9%			3.8%			n.s.

SD standard deviation, * *p* < 0.05, ** *p* < 0.01, *** *p* < 0.001, n.s. not significant. The denominator/number of patients n differs between rows because of missing values. ^#^ For neurological involvement, n = 81 corresponds to the number of patients who had documented neurological status, not to the whole cohort. Laboratory, electrocardiographic and echocardiographic parameters can be found in Supplementary Table S1. PD = primary diagnosis.

**Table 2:** Self-assessment regarding anxiety and depression.

Self-Assessment	Frequency	n
quality-of-life impairment due to amyloidosis		
yes	88.4%	112
mild	36.4%	99
moderate	40.4%	99
severe	23.2%	99
no	11.6%	112
change in anxiety since onset of amyloidosis-related symptoms		
more anxiety	54.5%	112
slightly increased	68.9%	61
moderately increased	27.9%	61
markedly increased	3.3%	61
no change	45.5%	112
quality-of-life impairment due to anxiety		
yes	42.5%	113
mild	54.2%	48
moderate	35.4%	48
severe	10.4%	48
no	57.5%	113
diagnosis of anxiety disorder	3.5%	113
anxiolytic drug	2.7%	113
psychological treatment	0.9%	113
history of anxiety disorder	0.9%	113
quality-of-life impairment due to depressed mood		
yes	58.4%	113
mild	68.2%	66
moderate	25.8%	66
severe	6.1%	66
no	41.6%	113
diagnosis of depression	2.7%	112
drugs for depression	2.7%	111
psychological treatment for depression	0.9%	111
history of depression	1.8%	112

**Table 3:** Results of the questionnaire-based assessment.

	Total Cohort				Anxiety and/or Depression								
					Yes				No				
	Mean		SD	n	Mean		SD	n	Mean		SD	n	*p*
**FACIT-Dyspnea questionnaire**													
Raw Dyspnea Score	11.67	±	9.09	109	18.53	±	8.55	29	9.18	±	7.98	80	***
Raw Functional Limitation Score	10.55	±	8.41	110	16.55	±	8.30	29	8.40	±	7.38	81	***
**FACIT-Fatigue questionnaire**													
Fatigue Subscale Score	33.02	±	12.03	109	20.79	±	8.95	29	37.45	±	9.72	80	***
severe fatigue	35.8%			109	75.9%			29	21.3%			80	***
**Distress thermometer**													
distress sum score	5.05	±	2.29	110	7.10	±	1.59	29	4.32	±	2.05	81	***
distress positive	62.7%			110	96.6%			29	50.6%			81	***
**GAD-7 questionnaire**													
GAD-7 sum score	5.25	±	4.45	110	10.62	±	4.31	29	3.33	±	2.50	81	***
no anxiety (GAD-7 0–4)	51.8%			110	10.3%			29	66.7%			81	***
mild anxiety (GAD-7 5–9)	30.0%			110	20.7%			29	33.3%			81	n.s.
moderate anxiety (GAD-7 10–14)	15.5%			110	58.6%			29	0.0%			81	***
severe anxiety (GAD-7 ≥ 15)	2.7%			110	10.3%			29	0.0%			81	*p* < 0.1
no impact on daily life (anxiety)	34.3%			108	3.4%			29	45.6%			79	***
mild limitation in daily life (anxiety)	44.4%			108	37.9%			29	46.8%			79	n.s.
relevant limitation in daily life (anxiety)	21.3%			108	58.6%			29	7.6%			79	***
**PHQ-9 questionnaire**													
PHQ-9 sum score	5.85	±	4.15	110	11.03	±	3.44	29	4.00	±	2.48	81	***
no impact on daily life (depression)	31.5%			108	0.0%			29	43.0%			79	***
mild limitation in daily life (depression)	50.0%			108	51.7%			29	49.4%			79	n.s.
relevant limitation in daily life (depression)	18.5%			108	48.3%			29	7.6%			79	***
minimal depressive symptoms (PHQ-9 0–4)	46.4%			110	3.4%			29	61.7%			81	***
mild depressive symptoms (PHQ-9 5–9)	34.5%			110	24.1%			29	38.3%			81	n.s.
moderate depression (PHQ-9 10–14)	13.6%			110	51.7%			29	0.0%			81	***
moderately severe depression (PHQ-9 15–19)	5.5%			110	20.7%			29	0.0%			81	**
severe depression (PHQ-9 ≥ 20)	0.0%			110	0.0%			29	0.0%			81	
major depression (categorical)	10.0%			110	37.9%			29	0.0%			81	***
other depression (categorical)	10.1%			109	31.0%			29	2.5%			80	**
**PA-F-KF questionnaire**													
PA-F-KF sum score	25.69	±	8.65	109	33.58	±	8.50	29	22.83	±	6.76	80	***
fear of progression positive screened	16.5%			109	48.3%			29	5.0%			80	***
**SF-36 questionnaire**													
Physical Component Summary (PCS)	36.92	±	9.84	109	31.49	±	7.91	28	38.79	±	9.79	81	***
Mental Component Summary (MCS)	46.81	±	9.06	109	37.50	±	6.05	28	50.03	±	7.59	81	***
Physical Functioning (PF)	49.24	±	27.35	110	36.72	±	26.40	29	53.72	±	26.43	81	**
Role limitations due to Physical health problems (RP)	47.02	±	33.88	109	31.25	±	24.18	28	52.47	±	35.15	81	***
Bodily Pain (BP)	61.53	±	30.58	110	43.45	±	31.41	29	68.00	±	27.71	81	***
General Health (GH)	44.41	±	17.75	110	28.78	±	13.91	29	50.00	±	15.53	81	***
Vitality (VT)	46.14	±	20.95	110	25.34	±	12.32	29	53.58	±	18.22	81	***
Social Functioning (SF)	68.75	±	24.46	110	45.26	±	19.88	29	77.16	±	20.14	81	***
Role limitations due to Emotional health problems (RE)	57.19	±	34.57	109	44.05	±	25.75	28	61.73	±	36.18	81	**
Mental Health (MH)	66.25	±	18.37	110	46.48	±	12.49	29	73.33	±	14.59	81	***

SD standard deviation, * *p* < 0.05, ** *p* < 0.01, *** *p* < 0.001, n.s. not significant.

**Table 4:** Comparison with the DEGS1 data set [30].

	AmyKoS Cohort				DEGS1 Data Set (75–79 Years)					
All	Mean		SD	n	Mean	SD	n	z	*p*-Value	
Physical Component Summary (PCS)	36.92	±	9.84	109	43.55	11.78	317	−5.76	<0.001	***
Mental Component Summary (MCS)	46.81	±	9.06	109	52.25	10.32	317	−5.21	<0.001	***
Physical Functioning (PF)	49.24	±	27.35	110	66.23	29.60	344	−5.56	<0.001	***
Role limitations due to Physical health problems (RP)	47.02	±	33.88	109	62.68	29.06	339	−4.34	<0.001	***
Bodily Pain (BP)	61.53	±	30.58	110	63.65	30.91	354	−0.63	0.526	n.s.
General Health (GH)	44.41	±	17.75	110	63.27	19.25	337	−9.47	<0.001	***
Vitality (VT)	46.14	±	20.95	110	61.42	21.59	341	−6.60	<0.001	***
Social Functioning (SF)	68.75	±	24.46	110	86.00	22.27	356	−6.60	<0.001	***
Role limitations due to Emotional health problems (RE)	57.19	±	34.57	109	79.01	27.18	332	−6.01	<0.001	***
Mental Health (MH)	66.25	±	18.37	110	76.09	17.57	339	−4.93	<0.001	***
**Depression (PHQ-9 ≥ 10)**										
Physical Component Summary (PCS)	31.11	±	8.31	20	33.15	13.61	19	−0.56	0.575	n.s.
Mental Component Summary (MCS)	38.05	±	6.74	20	40.38	10.38	19	−0.83	0.409	n.s.
General Health (GH)	27.18	±	13.95	21	40.36	17.59	20	−2.65	0.008	**
Physical Functioning (PF)	39.52	±	28.89	21	36.71	34.64	20	0.28	0.778	n.s.
Role limitations due to Physical health problems (RP)	31.25	±	25.49	20	40.54	35.47	19	−0.93	0.350	n.s.
Role limitations due to Emotional health problems (RE)	48.33	±	29.57	20	54.83	31.62	19	−0.66	0.508	n.s.
Social Functioning (SF)	42.86	±	21.86	21	56.33	25.09	20	−1.83	0.067	n.s.
Bodily Pain (BP)	41.91	±	33.62	21	42.02	30.49	20	−0.01	0.991	n.s.
Vitality (VT)	24.29	±	13.99	21	33.33	15.49	20	−1.96	0.050	n.s.
Mental Health (MH)	48.19	±	12.99	21	56.94	16.72	20	−1.87	0.062	n.s.

SD standard deviation, * *p* < 0.05, ** *p* < 0.01, *** *p* < 0.001, n.s. not significant.

**Table 5:** (**A**) Multivariate regression analysis of predictors of distress, anxiety and depression. (**B**) Robustness check using NYHA functional class and 6-min walk distance as surrogates for dyspnea and functional limitation.

**A**	**MCS**	**PCS**	**Distress**	**GAD-7** **Sum Score**	**PHQ-9** **Sum Score**
ECOG	0.06 [−3.13,3.25]	−1.29 [−4.07,1.49]	0.82 * [0.10,1.54]	1.06 [−0.44,2.57]	0.58 [−0.48,1.64]
Raw Dyspnea Score	−0.08 [−0.40,0.25]	−0.36 * [−0.64,−0.07]	0.10 ** [0.03,0.18]	0.03 [−0.12,0.19]	0.06 [−0.05,0.17]
Raw Functional Limitation Score	−0.08 [−0.47,0.31]	−0.28 [−0.62,0.07]	−0.00 [−0.09,0.09]	0.01 [−0.18,0.19]	0.05 [−0.08,0.18]
severe fatigue	−7.97 *** [−12.21,−3.73]	−0.07 [−3.77,3.63]	0.33 [−0.64,1.30]	2.43 * [0.40,4.45]	3.09 *** [1.67,4.52]
insomnia	−0.34 [−2.22,1.53]	−1.45 [−3.09,0.18]	0.24 [−0.19,0.67]	1.43 ** [0.54,2.32]	1.61 *** [0.98,2.23]
Gillmore stage	1.02 [−1.70,3.75]	0.42 [−1.96,2.79]	−0.31 [−0.92,0.30]	−0.36 [−1.64,0.92]	−0.74 [−1.64,0.16]
disease duration [a]	0.52 [−0.54,1.58]	−0.13 [−1.06,0.79]	0.01 [−0.23,0.25]	−0.28 [−0.78,0.22]	0.12 [−0.23,0.47]
age	0.28 [−0.04,0.60]	0.06 [−0.22,0.35]	−0.14 *** [−0.21,−0.07]	−0.14 [−0.30,0.01]	−0.04 [−0.15,0.07]
sex [% female]	−0.17 [−4.76,4.42]	−4.98 * [−8.98,−0.98]	−0.30 [−1.35,0.75]	0.91 [−1.28,3.10]	1.02 [−0.52,2.56]
Constant	27.28 * [2.23,52.33]	41.26 *** [19.42,63.11]	14.79 *** [9.11,20.46]	14.03 * [2.17,25.89]	5.22 [−3.12,13.56]
N	102.00	102.00	103.00	103.00	103.00
r2	0.26	0.49	0.39	0.32	0.61
Ninety-five percent confidence intervals in brackets. * *p* < 0.05, ** *p* < 0.01, *** *p* < 0.001.					
**B**	**MCS**	**PCS**	**Distress**	**GAD-7** **Sum Score**	**PHQ-9** **Sum Score**
ECOG	0.28 [−3.17,3.74]	−3.09 [−6.29,0.12]	0.88 * [0.13,1.64]	0.90 [−0.70,2.49]	0.76 [−0.39,1.91]
NYHA functional class	−2.71 [−6.18,0.75]	−4.44 ** [−7.65,−1.22]	0.68 [−0.10,1.45]	0.76 [−0.87,2.40]	1.22 * [0.04,2.40]
MWT distance	−0.01 [−0.03,0.01]	0.01 [−0.01,0.02]	−0.00 [−0.01,0.00]	−0.00 [−0.01,0.01]	−0.00 [−0.01,0.00]
severe fatigue	−8.06 *** [−12.28,−3.84]	−2.32 [−6.24,1.59]	0.28 [−0.67,1.23]	2.12 * [0.13,4.12]	3.27 *** [1.82,4.71]
insomnia	−0.55 [−2.49,1.40]	−3.26 ** [−5.07,−1.45]	0.54 * [0.11,0.98]	1.49 ** [0.58,2.41]	1.90 *** [1.24,2.56]
Gillmore stage	1.43 [−1.64,4.50]	0.57 [−2.28,3.42]	−0.45 [−1.12,0.23]	−0.60 [−2.01,0.82]	−1.11 * [−2.14,−0.09]
disease duration [a]	0.53 [−0.56,1.62]	−0.09 [−1.10,0.92]	−0.02 [−0.26,0.23]	−0.35 [−0.86,0.16]	0.10 [−0.27,0.47]
age	0.20 [−0.14,0.54]	0.10 [−0.21,0.42]	−0.16 *** [−0.24,−0.09]	−0.14 [−0.30,0.02]	−0.03 [−0.15,0.08]
sex [% female]	1.34 [−3.69,6.37]	−5.04 * [−9.71,−0.37]	−0.82 [−1.95,0.31]	0.60 [−1.78,2.98]	0.53 [−1.19,2.25]
Constant	39.98 * [8.97,70.99]	42.87 ** [14.10,71.65]	17.23 *** [10.35,24.10]	13.10 [−1.36,27.56]	3.64 [−6.81,14.10]
N	90.00	90.00	91.00	91.00	91.00
r2	0.24	0.44	0.40	0.29	0.60

Ninety-five percent confidence intervals in brackets. * *p* < 0.05, ** *p* < 0.01, *** *p* < 0.001.

## Data Availability

The data presented in this study cannot be shared publicly due to privacy regulations. The DEGS1 reference data are available from the Research Data Centre of the Robert Koch Institute as a Scientific Use File (https://doi.org/10.7797/16-200812-1-1-1) upon application.

## Supplementary Materials

The following supporting information can be downloaded at https://www.mdpi.com/article/10.3390/jcm15166348/s1: Table S1: Laboratory, electrocardiographic and echocardiographic characteristics of the study cohort, overall and stratified by a positive screening result for anxiety and/or depression; Table S2: Multivariate regression analysis (control for age and sex) of predictors of distress, anxiety, depression, fear of progression and quality of life impairment (MCS, PCS); Table S3: Sex-stratified predictor models. Linear regression of psychometric outcomes on the FACIT-Fatigue Fatigue Subscale Score and on ECOG performance status, adjusted for age; Table S4: Disease stage and functional disability. Linear regression of psychometric outcomes and SF-36 summary scales on NAC stage and on Coutinho stage, adjusted for age and sex; Table S5: Treatment status, treatment class and treatment duration. Linear regression of psychometric outcomes and SF-36 summary scales, adjusted for age and sex; Table S6: Sensitivity analysis: psychometric results in the full cohort and after exclusion of patients with a pre-existing anxiety disorder or depression or with chronic psychotropic medication; Table S7: Hierarchical regression models testing the incremental contribution of ATTR-specific features beyond markers of heart failure severity; Table S8: Sensitivity analysis of PA-F-KF scoring approaches in the 109 patients who completed at least ten of the twelve PA-F-KF items; Table S9: Multivariate regression analysis of coping strategies (control for age and sex).

## Abbreviations

The following abbreviations are used in this manuscript:

ATTR	Transthyretin amyloidosis
ATTRwt	Wild-type transthyretin amyloidosis
ATTRv	Variant (hereditary) transthyretin amyloidosis
AL	Light chain amyloidosis
_A_MY-NEED_S_	amyloidosis needs research and care program
AmyKoS	Amyloidosis cohort study at the Interdisciplinary Amyloidosis Center of Northern Bavaria
CI	Confidence interval
DEGS1	German Health Interview and Examination Survey for Adults
DPD	Diphosphono-propandicarboxylic acid
ECOG	Eastern Cooperative Oncology Group performance status
eGFR	estimated glomerular filtration rate
FACIT	Functional Assessment of Chronic Illness Therapy
GAD-7	7-item Generalized Anxiety Disorder scale
IQR	interquartile range
MCID	minimal clinically important difference
MCS	Mental Component Summary
NAC	National Amyloidosis Centre
NT-proBNP	N-terminal pro B-type natriuretic peptide
NYHA	New York Heart Association functional class
PA-F-KF	Fear of Progression Questionnaire, short form
PCS	Physical Component Summary
PHQ-9	9-item Patient Health Questionnaire
PRO	Patient-reported outcome
PROMIS	Patient-Reported Outcomes Measurement Information System
SD	standard deviation
SF-36	36-Item Short Form Health Survey
THAOS	Transthyretin Amyloidosis Outcomes Survey
TTR	transthyretin
6MWT	six-minute walk test
