# Supplementary material for: Psycho-Emotional Profile and Disease Burden in Wild-Type Transthyretin Amyloidosis
Source: J Clin Med. 2026 Aug 17;15(16):6348. doi: 10.3390/jcm15166348 (PMC13513315; doi:10.3390/jcm15166348)
Supplement: Supplementary file 1 [file jcm-15-06348-s001.zip › jcm-4472732-supplementary.pdf]

Journal of
Clinical Medicine

MDPI

Supplementary Materials

Psycho-Emotional Profile and Disease Burden in Wild-Type Transthyretin Amyloidosis

Ihne-Schubert, S.M. et al.

Table S1. Laboratory, electrocardiographic and echocardiographic characteristics of the study cohort, overall and stratified by a positive screening result for anxiety and/or depression.

	**Total cohort**				**Anxiety and/or depression**						
						**yes**			**no**		**p**
	Mean	±	*SD*	*n*	*Mean*	±	*SD*	*Mean*	±	*SD*	
Clinical findings											
Peripheral edema	35.7%			112	31.0%			36.3%			n.s.
jugular distention	43.4%			106	63.0%			35.5%			*
systolic blood pressure	137.18	±	19.64	106	129.18	±	14.86	140.47	±	20.40	**
diastolic blood pressure	81.06	±	15.62	106	79.43	±	9.93	81.79	±	17.32	n.s.
heart rate	69.50	±	13.38	106	71.37	±	14.70	68.66	±	12.63	n.s.
rhythm											
sinusrhythm	37.4%			107	31.0%			40.8%			n.s.
atrial fibrillation/flutter	46.7%			107	48.3%			46.1%			n.s.
pacemaker rhythm	15.0%			107	20.7%			11.8%			n.s.
Unknown rhythm	0.9%			107	0.0%			1.3%			n.s.
Laboratory parameters											
NT-proBNP	3,106.85	±	2950.22	113	3,728.79	±	2357.29	2,744.60	±	2651.17	p<0.1
troponin	52.74	±	34.00	113	72.94	±	41.53	44.50	±	25.67	***
eGFR	56.55	±	17.41	87	49.07	±	14.28	59.67	±	17.74	**
Echocardiography											
LVDd	44.81	±	6.28	112	43.69	±	6.15	45.23	±	6.36	n.s.
LVEF	52.72	±	10.36	113	53.59	±	8.79	52.36	±	10.84	n.s.
IVSd	18.89	±	3.06	113	19.41	±	3.29	18.68	±	2.97	n.s.
PWd	15.12	±	1.99	113	15.55	±	2.21	14.95	±	1.88	n.s.

https://doi.org//xxxxx

	**Total cohort**				**Anxiety and/or depression**						
					**yes**			**no**			**p**
	Mean	±	*SD*	*n*	*Mean*	±	*SD*	*Mean*	±	*SD*	
LVMI	172.03	±	41.39	106	176.70	±	47.63	170.05	±	39.61	n.s.
GLPS	-10.55	±	-3.59	112	-10.14	±	-3.50	-10.59	±	-3.56	n.s.
E/e′	16.73	±	6.89	111	18.87	±	6.19	16.03	±	7.09	*
tr-v_{max}	2.83	±	0.32	105	2.88	±	0.31	2.82	±	0.31	n.s.
stroke volume	73.75	±	20.43	112	69.52	±	24.49	75.27	±	18.94	n.s.
cardiac output	5.05	±	1.42	112	4.91	±	1.41	5.12	±	1.45	n.s.
TAPSE	15.74	±	5.19	105	14.77	±	4.08	16.10	±	5.30	n.s.
MAPSE	7.69	±	2.69	104	7.05	±	2.52	7.95	±	2.75	n.s.
Functional capacity											
6-MWT distance	321.26	±	121.96	99	262.98	±	112.95	337.31	±	119.25	**
6-MWT early termination	11.3%			97	21.7%			8.3%			n.s.

SD standard deviation; n number of patients with available data. eGFR estimated glomerular filtration rate; GLPS global longitudinal peak strain; IVSd interventricular septal thickness in diastole; LVDd left ventricular end-diastolic diameter; LVEF left ventricular ejection fraction; LVMI left ventricular mass index; MAPSE mitral annular plane systolic excursion; NT-proBNP N-terminal pro B-type natriuretic peptide; PWd posterior wall thickness in diastole; TAPSE tricuspid annular plane systolic excursion; tr-v_{max} peak tricuspid regurgitation velocity; 6-MWT six-minute walk test.

Table S2. Multivariate regression analysis (control for age and sex) of predictors of distress, anxiety, depression, fear of progression and quality of life impairment (MCS, PCS).

(A) Distress

		**ECOG**	**NYHA functional class**	**Raw Dyspnea Score**	**Raw Functional Limitation Score**	**MWT distance**	**Need for assistance (everyday activities)**	**Fatigue Subscale Score**	**Severe fatigue**	**insomnia**
age	-0.09*	-0.14***	-0.10**	-0.13***	-0.13***	-0.14***	-0.12**	-0.10**	-0.10**	-0.08*
	[-0.16,-0.01]	[-0.22,-0.06]	[-0.17,-0.03]	[-0.20,-0.07]	[-0.20,-0.06]	[-0.22,-0.07]	[-0.20,-0.05]	[-0.16,-0.04]	[-0.17,-0.03]	[-0.15,-0.01]
sex [% female]	-0.08	-0.45	-0.57	-0.33	-0.25	-0.96	-0.49	-0.24	-0.12	0.12
	[-1.29,1.13]	[-1.58,0.69]	[-1.72,0.57]	[-1.34,0.69]	[-1.30,0.81]	[-2.16,0.24]	[-1.65,0.66]	[-1.26,0.78]	[-1.24,1.00]	[-1.05,1.30]
variable		1.58***	1.50***	0.14***	0.13***	-0.01***	1.77***	-0.10***	1.76***	0.77**
		[0.93,2.23]	[0.83,2.17]	[0.09,0.18]	[0.09,0.18]	[-0.01,-0.00]	[0.90,2.64]	[-0.13,-0.07]	[0.89,2.64]	[0.34,1.19]
Constant	11.74***	15.42***	9.66**	13.91***	13.91***	19.09***	14.06***	16.43***	12.45***	10.20**
	[5.62,17.86]	[9.47,21.38]	[4.10,15.22]	[8.71,19.10]	[8.50,19.33]	[12.78,25.40]	[8.31,19.80]	[11.15,21.72]	[6.75,18.15]	[4.49,15.91]
N	98.00	105.00	110.00	97.00	98.00	97.00	96.00	109.00	98.00	110.00
r2	0.05	0.21	0.18	0.33	0.28	0.23	0.20	0.33	0.19	0.13

	**disease duration**	**Gillmore stage**	**NT-proBNP**	**troponin**	**eGFR**	**edema**	**jugular distention**	**tr-v_{max}**	**liver vein dilation**	**E/e'**
age	-0.07	-0.08*	-0.09*	-0.09*	-0.12**	-0.07	-0.09*	-0.08	-0.10*	-0.07
	[-0.16,0.01]	[-0.16,-0.00]	[-0.16,-0.01]	[-0.16,-0.01]	[-0.21,-0.03]	[-0.14,0.01]	[-0.17,-0.01]	[-0.16,0.00]	[-0.18,-0.01]	[-0.15,0.00]
sex [% female]	-0.08	-0.26	-0.18	0.04	-0.60	-0.09	-0.16	-0.27	-0.14	-0.26
	[-1.30,1.13]	[-1.51,0.98]	[-1.38,1.02]	[-1.16,1.25]	[-1.93,0.74]	[-1.32,1.14]	[-1.41,1.09]	[-1.54,1.01]	[-1.40,1.12]	[-1.49,0.97]
variable	0.11	0.41	0.00*	0.02*	-0.03*	-0.55	0.65	1.04	0.58	0.04
	[-0.17,0.39]	[-0.27,1.09]	[0.00,0.00]	[0.00,0.03]	[-0.06,-0.00]	[-1.46,0.36]	[-0.28,1.59]	[-0.45,2.53]	[-0.83,1.98]	[-0.03,0.10]
Constant	10.74**	10.83**	11.33***	11.13***	16.11***	10.65**	11.79***	8.37*	12.43***	10.19**
	[4.20,17.27]	[4.81,16.86]	[5.38,17.27]	[5.24,17.02]	[8.56,23.66]	[4.62,16.68]	[5.61,17.98]	[0.95,15.79]	[6.11,18.76]	[4.19,16.19]
N	97.00	110.00	110.00	110.00	84.00	109.00	93.00	102.00	95.00	108.00
r2	0.05	0.04	0.07	0.08	0.11	0.04	0.07	0.05	0.06	0.04

	**single**	**children**	**grand-children**	**worries not discussed**	**anxiety not discussed (not relevant)**
age	-0.06	-0.07	-0.07	-0.07	-0.07
	[-0.14,0.01]	[-0.15,0.00]	[-0.15,0.01]	[-0.15,0.01]	[-0.14,0.01]
sex [% female]	0.80	-0.21	-0.26	-0.15	-0.13
	[-0.67,2.26]	[-1.46,1.03]	[-1.53,1.01]	[-1.38,1.09]	[-1.35,1.09]
variable	-1.57*	-1.41	0.24	-0.75	-0.77
	[-2.85,-0.30]	[-4.02,1.20]	[-1.02,1.51]	[-2.20,0.69]	[-1.74,0.20]
Constant	10.26**	12.29***	10.62**	10.74**	10.65**
	[4.18,16.34]	[5.71,18.86]	[4.34,16.91]	[4.52,16.96]	[4.68,16.62]
N	97.00	107.00	104.00	109.00	110.00
r2	0.11	0.05	0.04	0.04	0.05

95% confidence intervals in brackets. * $p < 0.05$, ** $p < 0.01$, *** $p < 0.001$.

(B) Anxiety

		**ECOG**	**NYHA functional class**	**Raw Dyspnea Score**	**Raw Functional Limitation Score**	**MWT distance**	**need for assistance (everyday activities)**	**Fatigue Subscale Score**	**Severe fatigue**	**insomnia**
age	-0.06	-0.13	-0.07	-0.09	-0.11	-0.10	-0.11	-0.10	-0.09	-0.06
	[-0.20,0.09]	[-0.29,0.03]	[-0.22,0.08]	[-0.24,0.05]	[-0.25,0.03]	[-0.26,0.05]	[-0.25,0.02]	[-0.22,0.02]	[-0.22,0.05]	[-0.19,0.08]
sex	1.11	0.25	0.28	0.47	0.92	0.02	0.74	0.55	1.04	1.42
	[-1.08,3.31]	[-2.10,2.61]	[-2.12,2.68]	[-1.80,2.73]	[-1.17,3.01]	[-2.52,2.56]	[-1.43,2.92]	[-1.41,2.51]	[-0.99,3.08]	[-0.76,3.60]
variable		2.35**	1.61*	0.18***	0.16**	-0.01*	2.55**	-0.22***	3.21***	2.07***
		[1.01,3.70]	[0.20,3.01]	[0.09,0.27]	[0.06,0.26]	[-0.02,-0.00]	[0.91,4.19]	[-0.28,-0.16]	[1.62,4.79]	[1.28,2.86]
Constant	9.23	14.19*	6.74	10.56	11.80*	15.92*	12.74*	20.26***	10.52*	6.86
	[-1.89,20.34]	[1.83,26.55]	[-4.88,18.37]	[-0.63,21.74]	[1.09,22.50]	[2.59,29.25]	[1.94,23.55]	[10.08,30.43]	[0.17,20.87]	[-3.75,17.47]
N	98.00	105.00	110.00	109.00	98.00	97.00	96.00	109.00	98.00	110.00
r2	0.02	0.11	0.05	0.14	0.12	0.05	0.11	0.35	0.16	0.21
	**disease duration**	**Gillmore stage**	**NT-proBNP**	**troponin**	**eGFR**	**edema**	**jugular distention**	**tr-v_{max}**	**liver vein dilation**	**E/e′**

age	-0.03	-0.04	-0.04	-0.09	-0.10	-0.03	-0.04	-0.04	-0.04	-0.04
	[-0.18,0.13]	[-0.20,0.11]	[-0.20,0.11]	[-0.23,0.06]	[-0.26,0.06]	[-0.18,0.12]	[-0.19,0.11]	[-0.19,0.12]	[-0.19,0.11]	[-0.20,0.11]
sex	0.74	0.64	0.73	1.22	1.76	0.81	1.28	0.38	0.21	0.48
	[-1.69,3.17]	[-1.83,3.10]	[-1.69,3.15]	[-1.08,3.51]	[-0.71,4.23]	[-1.60,3.22]	[-1.24,3.80]	[-2.14,2.90]	[-2.25,2.68]	[-1.95,2.90]
variable	0.01	0.37	0.00	0.05***	-0.04	-1.38	1.02	1.73	1.51	0.09
	[-0.53,0.56]	[-0.97,1.71]	[-0.00,0.00]	[0.02,0.07]	[-0.09,0.01]	[-3.16,0.40]	[-0.79,2.83]	[-1.21,4.67]	[-0.95,3.97]	[-0.03,0.22]
Constant	7.44	7.94	8.13	9.45	15.01*	8.09	7.78	3.31	8.08	7.19
	[-4.94,19.82]	[-3.98,19.85]	[-3.81,20.08]	[-1.78,20.67]	[1.04,28.98]	[-3.73,19.91]	[-4.27,19.84]	[-11.36,17.97]	[-3.73,19.89]	[-4.63,19.01]
N	109.00	110.00	110.00	110.00	84.00	109.00	103.00	102.00	107.00	108.00
r2	0.00	0.01	0.01	0.12	0.06	0.03	0.02	0.02	0.02	0.02

	**single**	**children**	**grand-children**	**worries not discussed**	**anxiety not discussed (not relevant)**
age	-0.04	-0.04	-0.05	-0.04	-0.03
	[-0.20,0.11]	[-0.19,0.11]	[-0.21,0.11]	[-0.20,0.11]	[-0.17,0.11]
sex	0.04	0.79	0.74	0.67	0.71
	[-3.01,3.09]	[-1.71,3.29]	[-1.79,3.28]	[-1.74,3.07]	[-1.56,2.98]
variable	1.02	0.07	0.42	-2.23	-3.47***
	[-1.61,3.65]	[-5.19,5.32]	[-2.11,2.94]	[-5.05,0.59]	[-5.28,-1.66]
Constant	8.50	8.09	8.64	8.93	8.40
	[-3.62,20.61]	[-5.13,21.30]	[-3.89,21.18]	[-3.21,21.06]	[-2.74,19.55]
N	108.00	107.00	104.00	109.00	110.00
r2	0.01	0.01	0.01	0.03	0.12

95% confidence intervals in brackets. * $p < 0.05$, ** $p < 0.01$, *** $p < 0.001$.

(C) Depression

		ECOG	NYHA functional class	Raw Dyspnea Score	Raw Functional Limitation Score	MWT distance	Need for assistance (everyday activities	Fatigue Subscale Score	Severe fatigue	insomnia
age	0.03	-0.05	0.00	-0.04	-0.06	-0.04	-0.05	-0.03	-0.00	0.02
	[-0.11,0.17]	[-0.20,0.09]	[-0.13,0.14]	[-0.16,0.08]	[-0.18,0.07]	[-0.19,0.11]	[-0.18,0.08]	[-0.12,0.07]	[-0.11,0.11]	[-0.09,0.13]
sex	0.78	0.04	-0.08	0.20	0.47	-0.40	0.24	0.35	0.53	1.43
	[-1.40,2.97]	[-2.09,2.17]	[-2.25,2.09]	[-1.69,2.10]	[-1.42,2.37]	[-2.78,1.99]	[-1.82,2.29]	[-1.12,1.81]	[-1.22,2.27]	[-0.38,3.23]
variable		2.55***	2.26**	0.26***	0.25***	-0.01**	3.63***	-0.26***	5.47***	2.61***
		[1.33,3.77]	[0.99,3.53]	[0.18,0.33]	[0.17,0.34]	[-0.02,-0.00]	[2.08,5.18]	[-0.31,-0.22]	[4.17,6.76]	[1.95,3.26]
Constant	3.52	8.94	0.28	5.73	7.61	12.57*	8.23	16.68***	4.16	0.55
	[-7.56,14.60]	[-2.25,20.13]	[-10.21,10.77]	[-3.62,15.09]	[-2.07,17.29]	[0.05,25.08]	[-1.99,18.45]	[9.07,24.29]	[-4.43,12.74]	[-8.24,9.35]
N	98.00	105.00	110.00	109.00	98.00	97.00	96.00	109.00	109.00	110.00
r2	0.01	0.15	0.11	0.31	0.27	0.10	0.20	0.58	0.41	0.37

	Disease duration	Gillmore stage	NT-proBNP	troponin	eGFR	Edema	Jugular distention	tr-v_{max}	liver vein dilation	E/e′
age	0.06	0.04	0.04	0.01	-0.05	0.03	0.01	0.05	0.05	0.03
	[-0.09,0.20]	[-0.10,0.19]	[-0.10,0.18]	[-0.13,0.15]	[-0.21,0.10]	[-0.11,0.17]	[-0.13,0.15]	[-0.10,0.19]	[-0.09,0.19]	[-0.11,0.16]
sex	0.86	0.49	0.56	0.98	1.85	0.82	1.35	0.38	0.10	0.18
	[-1.32,3.04]	[-1.80,2.79]	[-1.69,2.80]	[-1.19,3.14]	[-0.53,4.23]	[-1.38,3.03]	[-0.91,3.61]	[-1.97,2.72]	[-2.20,2.41]	[-2.03,2.39]
variable	0.41	0.32	0.00	0.04**	-0.05*	-0.34	1.19	2.09	-0.27	0.16**
	[-0.09,0.91]	[-0.93,1.57]	[-0.00,0.00]	[0.01,0.06]	[-0.10,-0.00]	[-2.05,1.38]	[-0.50,2.88]	[-0.64,4.83]	[-2.57,2.03]	[0.05,0.28]
Constant	0.25	1.79	2.18	3.02	13.00	3.53	4.18	-3.89	1.88	1.04
	[-11.49,12.00]	[-9.32,12.89]	[-8.92,13.29]	[-7.58,13.63]	[-0.46,26.46]	[-7.64,14.71]	[-6.98,15.33]	[-17.52,9.74]	[-9.16,12.93]	[-9.69,11.78]
N	97.00	110.00	110.00	110.00	84.00	97.00	93.00	102.00	107.00	108.00
r2	0.03	0.01	0.02	0.10	0.08	0.01	0.04	0.03	0.01	0.08

	**single**	**children**	**grand-children**	**worries not discussed**	**anxiety not discussed (not relevant)**
age	0.06	0.05	0.04	0.04	0.06
	[-0.08,0.21]	[-0.09,0.19]	[-0.11,0.19]	[-0.10,0.18]	[-0.08,0.19]
sex	1.03	0.65	0.62	0.52	0.56
	[-1.80,3.87]	[-1.68,2.99]	[-1.74,2.99]	[-1.73,2.76]	[-1.57,2.68]
variable	-0.68	-0.17	0.46	-1.91	-3.17***
	[-3.13,1.77]	[-5.07,4.72]	[-1.89,2.82]	[-4.55,0.72]	[-4.86,-1.48]
Constant	0.95	2.15	2.37	2.86	2.22
	[-10.34,12.23]	[-10.16,14.46]	[-9.32,14.07]	[-8.47,14.19]	[-8.19,12.64]
N	108.00	107.00	104.00	109.00	110.00
r2	0.01	0.01	0.01	0.03	0.12

95% confidence intervals in brackets. * $p < 0.05$, ** $p < 0.01$, *** $p < 0.001$.

(D) Fear of Progression

		**ECOG**	**NYHA functional class**	**Raw Dyspnea Score**	**Raw Functional Limitation Score**	**MWT distance**	**Need for assistance (everyday activities**	**Fatigue Subscale Score**	**Severe fatigue**	**insomnia**
age	-0.00	-0.27	-0.12	-0.13	-0.19	-0.16	-0.10	-0.15	-0.06	-0.08
	[-0.30,0.30]	[-0.58,0.03]	[-0.41,0.18]	[-0.42,0.15]	[-0.47,0.08]	[-0.46,0.13]	[-0.40,0.20]	[-0.41,0.11]	[-0.34,0.23]	[-0.35,0.19]
sex	3.65	2.54	2.56	3.25	3.02	2.47	2.82	3.14	3.46	4.56*
	[-0.98,8.28]	[-1.97,7.06]	[-2.08,7.21]	[-1.10,7.60]	[-1.28,7.33]	[-2.35,7.29]	[-1.87,7.51]	[-0.98,7.26]	[-0.89,7.81]	[0.20,8.92]
variable		4.47**	3.10*	0.35***	0.41***	-0.02*	4.17*	-0.34***	6.31***	3.36***
		[1.89,7.05]	[0.37,5.82]	[0.16,0.53]	[0.23,0.60]	[-0.03,-0.00]	[0.61,7.73]	[-0.46,-0.22]	[2.90,9.73]	[1.77,4.94]
Constant	25.01*	44.74***	27.07*	31.38**	36.25**	42.91**	30.75*	48.28***	27.34*	27.50*
	[1.57,48.46]	[21.04,68.44]	[4.60,49.54]	[9.16,53.59]	[14.91,57.59]	[17.59,68.23]	[7.47,54.03]	[26.92,69.65]	[5.27,49.41]	[6.25,48.75]
N	97.00	105.00	109.00	96.00	109.00	96.00	95.00	108.00	97.00	109.00
r2	0.03	0.12	0.07	0.15	0.17	0.06	0.08	0.24	0.15	0.16

	disease duration	Gillmore stage	NT-proBNP	troponin	eGFR	edema	Jugular distention	tr-v_{max}	liver vein dilation	E/e′
age	0.02	-0.06	-0.07	-0.15	-0.06	-0.05	-0.07	-0.07	-0.04	-0.07
	[-0.30,0.33]	[-0.36,0.24]	[-0.37,0.22]	[-0.43,0.13]	[-0.39,0.27]	[-0.34,0.24]	[-0.36,0.22]	[-0.37,0.23]	[-0.33,0.26]	[-0.36,0.22]
sex	3.71	3.41	3.44	4.37	5.87*	3.67	5.10*	3.53	2.00	2.84
	[-0.98,8.39]	[-1.36,8.18]	[-1.23,8.10]	[-0.08,8.82]	[0.79,10.95]	[-1.00,8.34]	[0.36,9.85]	[-1.26,8.32]	[-2.60,6.60]	[-1.80,7.48]
variable	0.22	0.35	0.00	0.09**	-0.07	-0.53	2.60	1.49	1.35	0.20
	[-0.86,1.30]	[-2.25,2.95]	[-0.00,0.00]	[0.04,0.13]	[-0.18,0.04]	[-4.01,2.95]	[-0.82,6.02]	[-4.13,7.11]	[-3.96,6.66]	[-0.04,0.44]
Constant	23.35	29.16*	30.17*	32.29**	34.14*	28.93*	29.20*	26.44	27.89*	27.77*
	[-1.84,48.54]	[6.11,52.20]	[7.12,53.21]	[10.49,54.08]	[5.42,62.86]	[6.01,51.85]	[6.51,51.89]	[-1.52,54.40]	[4.82,50.96]	[5.17,50.37]
N	96.00	109.00	109.00	109.00	83.00	108.00	102.00	101.00	94.00	107.00
r2	0.03	0.02	0.03	0.12	0.08	0.02	0.06	0.03	0.01	0.05

	single	children	grand-children	worries not discussed	anxiety not discussed (not relevant)
age	-0.04	-0.05	-0.04	-0.04	-0.05
	[-0.34,0.27]	[-0.34,0.24]	[-0.34,0.26]	[-0.33,0.26]	[-0.31,0.21]
sex	1.68	3.20	3.36	3.33	3.46
	[-4.09,7.46]	[-1.60,8.00]	[-1.43,8.15]	[-1.25,7.91]	[-0.72,7.65]
variable	2.69	4.39	3.58	-5.96*	-8.73***
	[-2.34,7.73]	[-5.67,14.45]	[-1.20,8.35]	[-11.34,-0.59]	[-12.11,-5.36]
Constant	27.48*	25.18	25.03*	28.57*	31.22**
	[3.48,51.48]	[-0.17,50.53]	[1.32,48.74]	[5.45,51.68]	[10.69,51.76]
N	96.00	106.00	104.00	108.00	109.00
r2	0.03	0.03	0.04	0.07	0.22

95% confidence intervals in brackets. * $p < 0.05$, ** $p < 0.01$, *** $p < 0.001$.

(E) Quality of Life – MCS

		ECOG	NYHA functional class	Raw Dyspnea Score	Raw Functional Limitation Score	MWT distance	Need for assistance (everyday activities)	Fatigue Subscale Score	Severe fatigue	insomnia
age	0.21	0.25	0.25	0.29	0.30*	0.28	0.32*	0.28*	0.26	0.19
	[-0.05,0.46]	[-0.08,0.58]	[-0.06,0.56]	[-0.01,0.59]	[0.00,0.60]	[-0.05,0.61]	[0.02,0.62]	[0.02,0.54]	[-0.01,0.53]	[-0.11,0.49]
sex	1.84	0.50	0.91	0.32	0.31	1.99	1.35	0.28	-0.06	-0.93
	[-2.07,5.75]	[-4.38,5.37]	[-3.95,5.77]	[-4.34,4.97]	[-4.31,4.93]	[-3.35,7.32]	[-3.41,6.12]	[-3.80,4.37]	[-4.37,4.24]	[-5.72,3.85]
variable		-2.89*	-3.66*	-0.36***	-0.39***	0.01	-6.65***	0.43***	-9.34***	-2.41**
		[-5.71,-0.06]	[-6.50,-0.82]	[-0.54,-0.17]	[-0.59,-0.19]	[-0.01,0.02]	[-10.10,-3.21]	[0.31,0.56]	[-12.55,-6.13]	[-4.17,-0.65]
Constant	34.19***	28.19*	35.67**	27.87*	27.26*	22.26	24.32*	10.11	29.82**	34.96**
	[14.37,54.01]	[2.61,53.76]	[11.97,59.38]	[4.71,51.03]	[4.24,50.27]	[-5.82,50.33]	[1.21,47.44]	[-11.13,31.34]	[8.50,51.14]	[11.38,58.54]
N	98.00	104.00	109.00	108.00	109.00	96.00	106.00	108.00	108.00	109.00
r2	0.04	0.05	0.07	0.13	0.13	0.04	0.14	0.33	0.25	0.08

	disease duration	Gillmore stage	NT-proBNP	troponin	eGFR	edema	Jugular distention	tr-v_{max}	liver vein dilation	E/e′
Age	0.17	0.20	0.22	0.29	0.31	0.17	0.24	0.21	0.19	0.19
	[-0.15,0.49]	[-0.12,0.52]	[-0.10,0.53]	[-0.00,0.58]	[-0.06,0.68]	[-0.14,0.48]	[-0.07,0.55]	[-0.11,0.54]	[-0.11,0.50]	[-0.12,0.50]
sex	-0.20	0.04	-0.08	-1.21	-0.77	-0.39	-0.81	0.30	0.89	0.36
	[-5.15,4.75]	[-4.96,5.05]	[-4.96,4.81]	[-5.79,3.36]	[-6.44,4.90]	[-5.29,4.51]	[-5.81,4.18]	[-4.81,5.41]	[-4.10,5.88]	[-4.57,5.29]
variable	0.00	-0.80	-0.00	-0.11***	0.10	0.96	-4.27*	-1.62	-1.19	-0.18
	[-1.11,1.12]	[-3.54,1.94]	[-0.00,0.00]	[-0.16,-0.06]	[-0.02,0.22]	[-2.68,4.59]	[-7.87,-0.67]	[-7.58,4.34]	[-6.18,3.79]	[-0.43,0.08]
Constant	33.41*	32.54*	31.14*	29.80*	16.75	33.22**	30.07*	34.29*	31.77*	34.27**
	[7.82,59.01]	[8.07,57.02]	[6.81,55.48]	[7.23,52.36]	[-15.36,48.86]	[8.98,57.47]	[5.94,54.20]	[4.42,64.15]	[7.63,55.91]	[10.08,58.45]
N	108.00	109.00	109.00	109.00	84.00	108.00	102.00	101.00	106.00	107.00
r2	0.01	0.01	0.03	0.16	0.05	0.01	0.07	0.02	0.02	0.03

	single	children	grand-children	worries not discussed	anxiety not discussed (not relevant)
age	0.16	0.19	0.24	0.22	0.17
	[-0.16,0.48]	[-0.12,0.50]	[-0.08,0.56]	[-0.10,0.53]	[-0.14,0.47]
sex	0.00	0.17	0.31	-0.01	-0.15
	[-6.20,6.20]	[-4.90,5.24]	[-4.72,5.35]	[-4.90,4.87]	[-4.98,4.68]
variable	0.57	0.65	0.92	4.10	3.96*
	[-4.79,5.93]	[-9.97,11.27]	[-4.09,5.94]	[-1.63,9.83]	[0.11,7.81]
Constant	33.92**	31.35*	27.06*	29.25*	32.66**
	[9.00,58.85]	[4.38,58.32]	[1.91,52.22]	[4.40,54.11]	[8.76,56.56]
N	107.00	106.00	103.00	108.00	109.00
r2	0.01	0.01	0.02	0.03	0.05

95% confidence intervals in brackets. * $p < 0.05$, ** $p < 0.01$, *** $p < 0.001$.

(F) Quality of Life – PCS

		ECOG	NYHA functional class	Raw Dyspnea Score	Raw Functional Limitation Score	MWT distance	Need for assistance (everyday activities)	Fatigue Subscale Score	Severe fatigue	insomnia
age	-0.21	0.05	-0.07	-0.01	0.02	0.05	-0.05	-0.11	-0.15	-0.20
	[-0.54,0.11]	[-0.28,0.37]	[-0.37,0.23]	[-0.26,0.25]	[-0.24,0.28]	[-0.28,0.39]	[-0.35,0.25]	[-0.38,0.16]	[-0.45,0.15]	[-0.50,0.11]
sex	-5.61*	-4.44	-3.67	-4.72*	-4.89*	-4.64	-4.96*	-5.29*	-5.67*	-7.02**
	[-10.65,-0.57]	[-9.20,0.32]	[-8.39,1.04]	[-8.72,-0.72]	[-8.91,-0.88]	[-10.07,0.79]	[-9.80,-0.12]	[-9.56,-1.01]	[-10.38,-0.96]	[-11.87,-2.18]
variable		-6.03***	-7.22***	-0.68***	-0.75***	0.03***	-8.69***	0.45***	-8.18***	-3.74***
		[-8.78,-3.27]	[-9.98,-4.47]	[-0.84,-0.52]	[-0.92,-0.57]	[0.02,0.05]	[-12.19,-5.18]	[0.32,0.58]	[-11.69,-4.66]	[-5.53,-1.96]
Constant	54.51***	36.49**	60.08***	45.81***	43.86***	23.02	45.21***	31.24**	52.40***	57.99***
	[28.97,80.04]	[11.54,61.45]	[37.09,83.06]	[25.91,65.71]	[23.84,63.88]	[-5.58,51.62]	[21.72,68.71]	[9.01,53.46]	[29.06,75.73]	[34.15,81.83]
N	98.00	104.00	109.00	108.00	109.00	96.00	106.00	108.00	108.00	109.00
r2	0.07	0.21	0.26	0.44	0.44	0.19	0.25	0.36	0.23	0.20

	disease duration	Gillmore stage	NT-proBNP	troponin	eGFR	edema	jugular distention	tr-v_{max}	liver vein dilation	E/e′
age	-0.29	-0.13	-0.16	-0.19	-0.16	-0.23	-0.19	-0.22	-0.21	-0.20
	[-0.62,0.04]	[-0.46,0.20]	[-0.48,0.17]	[-0.52,0.14]	[-0.53,0.21]	[-0.55,0.10]	[-0.52,0.13]	[-0.56,0.13]	[-0.54,0.12]	[-0.53,0.12]
sex	-5.85*	-4.88	-5.70*	-6.24*	-6.10*	-6.06*	-6.27*	-5.36	-6.72*	-5.23*
	[-10.94,-0.76]	[-10.05,0.30]	[-10.81,-0.60]	[-11.42,-1.05]	[-11.75,-0.46]	[-11.24,-0.89]	[-11.56,-0.99]	[-10.75,0.02]	[-12.08,-1.35]	[-10.40,-0.05]
variable	-0.89	-3.07*	-0.00	-0.03	0.14*	2.31	-3.81	-4.87	-2.53	-0.20
	[-2.04,0.26]	[-5.91,-0.23]	[-0.00,-0.00]	[-0.09,0.02]	[0.02,0.26]	[-1.52,6.15]	[-7.62,0.00]	[-11.15,1.41]	[-7.89,2.83]	[-0.47,0.06]
Constant	61.96***	52.39***	52.22***	54.40***	42.25*	54.92***	54.64***	68.57***	54.65***	56.87***
	[35.65,88.28]	[27.08,77.71]	[26.78,77.66]	[28.80,80.01]	[10.29,74.21]	[29.31,80.54]	[29.11,80.18]	[37.08,100.06]	[28.67,80.62]	[31.47,82.26]
N	108.00	109.00	109.00	109.00	84.00	108.00	102.00	101.00	106.00	107.00
r2	0.09	0.11	0.10	0.08	0.14	0.08	0.11	0.09	0.08	0.09

	single	children	grand-children	worries not discussed	anxiety not discussed (not relevant)
age	-0.25	-0.20	-0.21	-0.19	-0.23
	[-0.59,0.08]	[-0.52,0.12]	[-0.54,0.13]	[-0.53,0.14]	[-0.55,0.09]
sex [% fe-male]	-7.93*	-6.94**	-6.53*	-5.79*	-5.85*
	[-14.38,-1.47]	[-12.15,-1.73]	[-11.94,-1.12]	[-10.99,-0.59]	[-10.96,-0.75]
variable	2.22	13.41*	1.96	2.71	3.94
	[-3.36,7.81]	[2.50,24.33]	[-3.43,7.35]	[-3.38,8.81]	[-0.14,8.01]
Constant	57.39***	40.65**	52.19***	52.73***	54.80***
	[31.44,83.35]	[12.93,68.37]	[25.16,79.22]	[26.29,79.18]	[29.53,80.07]
N	107.00	106.00	103.00	108.00	109.00
r2	0.08	0.13	0.08	0.07	0.10

95% confidence intervals in brackets. * $p < 0.05$, ** $p < 0.01$, *** $p < 0.001$.

Table S3. Sex-stratified predictor models.Linear regression of psychometric outcomes on the FACIT-Fatigue Fatigue Subscale Score and on ECOG performance status, adjusted for age.

Outcome	Predictor	Men: b (p)	Women: b (p)
GAD-7 sum score	FACIT-Fatigue Fatigue Subscale Score	−0.23 (<0.001)	−0.23 (0.023)
GAD-7 sum score	ECOG performance status	+2.19 (0.005)	+3.35 (0.048)
PHQ-9 sum score	FACIT-Fatigue Fatigue Subscale Score	−0.25 (<0.001)	−0.38 (<0.001)
PHQ-9 sum score	ECOG performance status	+1.89 (0.007)	+5.37 (0.001)
Distress Thermometer	FACIT-Fatigue Fatigue Subscale Score	−0.10 (<0.001)	−0.11 (0.046)
Distress Thermometer	ECOG performance status	+1.49 (<0.001)	+1.62 (0.074)
Physical component summary	FACIT-Fatigue Fatigue Subscale Score	+0.47 (<0.001)	+0.27 (0.185)
Physical component summary	ECOG performance status	−5.78 (<0.001)	−6.07 (0.056)
Mental component summary	FACIT-Fatigue Fatigue Subscale Score	+0.44 (<0.001)	+0.46 (0.050)
Mental component summary	ECOG performance status	−2.64 (0.094)	−3.90 (0.336)

b regression coefficient. Models were fitted separately in men (n = 94) and women (n = 16) and adjusted for age. The predictor structure is concordant across sexes. Point estimates are larger in women, but confidence intervals overlap throughout and the small number of women limits precision.

Table S4. Disease stage and functional disability. Linear regression of psychometric outcomes and SF-36 summary scales on NAC stage and on Coutinho stage, adjusted for age and sex.

**Outcome**	**NAC stage II–III vs. I**		**Coutinho stage II vs. I**	
	**b**	**95% CI**	**b**	**95% CI**
GAD-7 sum score	1.02	[-0.77, 2.81]	1.74	[-0.93, 4.41]
PHQ-9 sum score	0.64	[-1.04, 2.31]	2.60*	[0.14, 5.05]
Distress Thermometer	0.59	[-0.32, 1.49]	0.71	[-0.65, 2.07]
PA-F-KF sum score	2.22	[-1.24, 5.68]	4.07	[-1.06, 9.21]
Physical component summary	-3.55	[-7.37, 0.27]	-5.83*	[-11.49, -0.16]
Mental component summary	-1.57	[-5.24, 2.10]	-3.47	[-8.90, 1.97]

*b regression coefficient; CI confidence interval. NAC stage II–III was compared with stage I, and Coutinho stage II (need for a walking aid) with stage I. n = 109 to 110. * p < 0.05.*

Table S5. Treatment status, treatment class and treatment duration. Linear regression of psychometric outcomes and SF-36 summary scales, adjusted for age and sex.

**Outcome**	**Any treatment vs. none**		**Treatment at survey**		**Duration (per year)**	
	**b**	**95% CI**	**b**	**95% CI**	**b**	**95% CI**
Distress Thermometer	-0.55	[-2.46, 1.37]	-0.41	[-1.47, 0.66]	0.49	[-0.02, 1.00]
GAD-7 sum score	-2.57	[-6.30, 1.17]	-1.28	[-3.37, 0.80]	0.27	[-0.79, 1.33]
PHQ-9 sum score	-0.72	[-4.23, 2.79]	-0.03	[-1.99, 1.93]	0.08	[-0.84, 1.00]
PA-F-KF sum score	-3.93	[-11.17, 3.31]	-1.04	[-5.10, 3.02]	0.35	[-1.51, 2.20]
FACIT-Fatigue Fatigue Subscale Score	-4.72	[-14.79, 5.35]	-0.32	[-5.96, 5.32]	-0.47	[-3.30, 2.36]
Physical component summary	0.81	[-7.27, 8.89]	1.67	[-2.83, 6.18]	-2.39*	[-4.37, -0.41]
Mental component summary	0.96	[-6.70, 8.62]	1.48	[-2.79, 5.75]	1.33	[-0.74, 3.40]

**Treatment class, reference: no disease-modifying treatment**	**TTR stabilizer alone**		**TTR stabilizer plus trial medication**	
	**b**	**95% CI**	**b**	**95% CI**
Distress Thermometer	-0.50	[-2.43, 1.44]	-0.73	[-2.84, 1.37]
GAD-7 sum score	-2.46	[-6.24, 1.32]	-2.96	[-7.07, 1.15]
PHQ-9 sum score	-0.69	[-4.25, 2.86]	-0.80	[-4.67, 3.06]
PA-F-KF sum score	-3.78	[-11.10, 3.55]	-4.54	[-12.55, 3.47]
FACIT-Fatigue Fatigue Subscale Score	-4.96	[-15.14, 5.22]	-3.85	[-14.92, 7.22]
Physical component summary	0.88	[-7.30, 9.06]	0.56	[-8.34, 9.45]
Mental component summary	0.98	[-6.77, 8.74]	0.87	[-7.55, 9.30]

*b regression coefficient; CI confidence interval. Seven patients received no disease-modifying therapy; all treated patients received a TTR stabilizer, of whom 25 were simultaneously enrolled in a phase III trial (HELIOS-B, CARDIO-TTRansform) and additionally received blinded study medication. No patient was exposed to a gene-silencing agent outside a trial. Treatment duration refers to the stabilizer and was derivable for 72 patients. n = 71 to 110. * p < 0.05.*

Table S6. Sensitivity analysis: psychometric results in the full cohort and after exclusion of patients with a pre-existing anxiety disorder or depression or with chronic psychotropic medication.

	Full cohort (n = 110)	Excluding 5 patients (n = 105)
GAD-7 sum score, mean ± SD	5.25 ± 4.45	5.12 ± 4.37
PHQ-9 sum score, mean ± SD	5.85 ± 4.15	5.77 ± 4.10
Distress Thermometer, mean ± SD	5.05 ± 2.29	4.98 ± 2.29
GAD-7 ≥ 10 points	18.2%	17.1%
PHQ-9 ≥ 10 points	19.1%	19.0%
Anxiety and/or depression	26.4%	25.7%

A pre-existing disorder was defined as an anxiety disorder or depression with onset before the manifestation of amyloidosis (n = 2); chronic psychotropic medication comprised anxiolytics (n = 3) and antidepressants (n = 2). Multivariable models were unchanged in sign and significance after exclusion.

Table S7. Hierarchical regression models testing the incremental contribution of ATTR-specific features beyond markers of heart failure severity.

Outcome	R^2 heart failure model	R^2 adding ATTR-specific features	ΔR^2	F	p
GAD-7 sum score	0.129	0.179	0.051	0.83	0.511
PHQ-9 sum score	0.176	0.272	0.096	1.77	0.148
Distress Thermometer	0.356	0.416	0.060	1.39	0.249
FACIT-Fatigue Fatigue Subscale Score	0.300	0.370	0.069	1.49	0.219

The base model comprised ECOG performance status, log NT-proBNP, left ventricular ejection fraction, six-minute walk distance, age and sex. ATTR-specific features added to the model were neurological involvement, Coutinho stage, carpal tunnel syndrome and NAC stage. All models were fitted in the same analysis sample (n = 58). ATTR-specific features contributed significantly only to the explanation of fatigue.

Table S8. Sensitivity analysis of PA-F-KF scoring approaches in the 109 patients who completed at least ten of the twelve PA-F-KF items.

Scoring approach	Mean ± SD	Positive (≥ 34 points), n (%)
Extrapolated 12-item score (primary analysis)	25.69 ± 8.65	18 (16.5)
Ten universally applicable items, rescaled to the 12-item metric	26.87 ± 8.96	22 (20.2)
Instrument-conform: lowest value for non-applicable items	24.15 ± 7.78	15 (13.8)

Classification agreed in 93.6% to 97.2% of patients across the three approaches, and the scores correlated closely (r ≥ 0.987). The original instruction of the PA-F-KF directs respondents to select the lowest response category for items that do not apply; the third approach implements this convention. The prevalence estimate is therefore robust to the handling of non-applicable items.

Table S9. Multivariate regression analysis of coping strategies (control for age and sex).

DT	physical activity	creative activity	spiritual activity	social activity	cultural activity	cosy activity	relax
Age	-0.11**	-0.08*	-0.09*	-0.09*	-0.10*	-0.08*	-0.09*
	[-0.18,-0.04]	[-0.16,-0.00]	[-0.16,-0.01]	[-0.17,-0.01]	[-0.18,-0.02]	[-0.16,-0.01]	[-0.16,-0.01]
sex	-0.32	-0.08	-0.08	-0.05	-0.12	-0.08	0.07
	[-1.44,0.80]	[-1.30,1.14]	[-1.29,1.13]	[-1.25,1.16]	[-1.32,1.09]	[-1.29,1.13]	[-1.15,1.29]
variable	-0.94***	0.14	-0.30	-0.34	-0.44	0.18	-0.43
	[-1.38,-0.50]	[-0.43,0.70]	[-0.79,0.18]	[-1.00,0.31]	[-1.09,0.21]	[-0.47,0.84]	[-1.04,0.18]
Constant	16.05***	11.26***	12.25***	12.91***	13.62***	11.09**	12.33***
	[10.06,22.03]	[4.74,17.77]	[6.06,18.44]	[6.39,19.43]	[6.93,20.30]	[4.53,17.66]	[6.18,18.47]
N	98.00	97.00	97.00	98.00	98.00	98.00	98.00
r2	0.20	0.05	0.06	0.06	0.07	0.05	0.07

GAD-7	physical activity	creative activity	spiritual activity	social activity	cultural activity	cosy activity	relax
age	-0.10	-0.07	-0.06	-0.07	-0.09	-0.06	-0.06
	[-0.23,0.03]	[-0.22,0.07]	[-0.20,0.09]	[-0.21,0.07]	[-0.24,0.06]	[-0.20,0.09]	[-0.20,0.09]
sex	0.67	1.15	1.12	1.23	1.03	1.12	1.33
	[-1.35,2.69]	[-1.05,3.34]	[-1.10,3.33]	[-0.93,3.40]	[-1.14,3.21]	[-1.09,3.32]	[-0.90,3.55]
Variable	-1.76***	-0.68	-0.19	-1.15	-1.01	0.21	-0.62
	[-2.55,-0.96]	[-1.70,0.35]	[-1.08,0.70]	[-2.33,0.02]	[-2.18,0.16]	[-0.98,1.41]	[-1.72,0.49]
Constant	17.26**	11.69	9.56	13.18*	13.53*	8.47	10.07
	[6.45,28.06]	[-0.05,23.42]	[-1.77,20.89]	[1.51,24.85]	[1.45,25.60]	[-3.47,20.40]	[-1.14,21.28]
N	98.00	97.00	97.00	98.00	98.00	98.00	98.00
r2	0.18	0.03	0.02	0.05	0.05	0.02	0.03

PHQ-9	physical activity	creative activity	spiritual activity	social activity	cultural activity	cosy activity	relax
age	-0.03	0.02	0.03	0.02	-0.02	0.03	0.03
	[-0.15,0.10]	[-0.12,0.16]	[-0.12,0.17]	[-0.12,0.16]	[-0.16,0.13]	[-0.12,0.17]	[-0.11,0.17]
sex	0.24	0.80	0.79	0.83	0.68	0.77	1.00
	[-1.67,2.15]	[-1.41,3.01]	[-1.42,3.00]	[-1.36,3.02]	[-1.47,2.83]	[-1.41,2.96]	[-1.22,3.21]
variable	-2.13***	-0.27	0.27	-0.47	-1.25*	-0.61	-0.62
	[-2.88,-1.38]	[-1.30,0.76]	[-0.62,1.16]	[-1.65,0.72]	[-2.41,-0.10]	[-1.79,0.57]	[-1.73,0.48]
Constant	13.27*	4.52	3.09	5.11	8.85	5.67	4.37
	[3.05,23.49]	[-7.27,16.30]	[-8.19,14.37]	[-6.71,16.94]	[-3.08,20.78]	[-6.16,17.51]	[-6.80,15.54]
N	98.00	97.00	97.00	98.00	98.00	98.00	98.00
r2	0.26	0.01	0.01	0.01	0.05	0.02	0.02

PA-F	physical activity	creative activity	spiritual activity	social activity	cultural activity	cosy activity	relax
age	-0.05	-0.04	0.00	-0.00	-0.10	0.00	-0.00
	[-0.35,0.25]	[-0.34,0.26]	[-0.30,0.30]	[-0.30,0.30]	[-0.40,0.21]	[-0.30,0.30]	[-0.30,0.29]
sex	3.17	3.71	3.65	3.68	3.44	3.66	4.34
	[-1.39,7.73]	[-0.89,8.31]	[-0.99,8.29]	[-0.98,8.34]	[-1.09,7.97]	[-0.98,8.31]	[-0.32,9.00]
variable	-1.94*	-1.55	-0.66	-0.28	-2.81*	0.74	-1.93
	[-3.74,-0.15]	[-3.69,0.59]	[-2.53,1.20]	[-2.81,2.24]	[-5.25,-0.36]	[-1.77,3.25]	[-4.26,0.40]
Constant	33.94**	30.59*	26.11*	25.98*	37.01**	22.39	27.72*
	[9.49,58.38]	[6.03,55.15]	[2.40,49.82]	[0.86,51.11]	[11.80,62.22]	[-2.74,47.53]	[4.26,51.19]
N	97.00	97.00	97.00	97.00	97.00	97.00	97.00
r2	0.07	0.05	0.03	0.03	0.08	0.03	0.05

MCS SF-36	physical activity	creative activity	spiritual activity	social activity	cultural activity	cosy activity	relax
age	0.22	0.23	0.21	0.21	0.21	0.21	0.21
	[-0.04,0.47]	[-0.03,0.48]	[-0.05,0.46]	[-0.05,0.46]	[-0.05,0.48]	[-0.05,0.46]	[-0.05,0.46]
sex	1.94	1.75	1.77	1.83	1.86	1.84	1.52
	[-2.01,5.88]	[-2.19,5.68]	[-2.18,5.72]	[-2.10,5.77]	[-2.08,5.79]	[-2.09,5.77]	[-2.46,5.49]
variable	0.40	0.64	0.08	0.03	0.25	0.28	0.93
	[-1.15,1.95]	[-1.20,2.47]	[-1.51,1.66]	[-2.10,2.17]	[-1.87,2.37]	[-1.84,2.41]	[-1.05,2.91]
Constant	32.37**	31.62**	33.79**	34.07**	33.11**	33.19**	32.92**
	[11.24,53.49]	[10.60,52.63]	[13.62,53.96]	[12.84,55.30]	[11.25,54.98]	[11.90,54.48]	[12.90,52.94]
N	98.00	97.00	97.00	98.00	98.00	98.00	98.00
r2	0.04	0.04	0.04	0.04	0.04	0.04	0.05

PCS SF-36	physical activity	creative activity	spiritual activity	social activity	cultural activity	cosy activity	relax
age	-0.13	-0.21	-0.23	-0.19	-0.14	-0.22	-0.23
	[-0.43,0.17]	[-0.54,0.12]	[-0.56,0.10]	[-0.52,0.13]	[-0.47,0.19]	[-0.55,0.11]	[-0.55,0.10]
sex	-4.89*	-5.87*	-5.90*	-6.32*	-5.58*	-5.95*	-6.18*
	[-9.63,-0.14]	[-11.07,-0.67]	[-11.11,-0.70]	[-11.40,-1.24]	[-10.68,-0.49]	[-11.14,-0.76]	[-11.39,-0.97]
variable	4.26***	0.68	-0.36	3.06*	2.48	0.50	1.24
	[2.45,6.07]	[-1.65,3.02]	[-2.43,1.71]	[0.33,5.79]	[-0.18,5.14]	[-2.08,3.08]	[-1.22,3.71]
Constant	36.96**	52.97***	56.60***	45.06**	44.83**	53.56***	53.72***
	[12.29,61.64]	[26.06,79.88]	[30.68,82.52]	[18.02,72.10]	[17.19,72.47]	[25.99,81.12]	[28.04,79.41]
N	109.00	107.00	106.00	108.00	108.00	109.00	108.00
r2	0.23	0.07	0.07	0.11	0.10	0.07	0.08

95% confidence intervals in brackets. * $p < 0.05$, ** $p < 0.01$, *** $p < 0.001$.
